# Comprehensive Analysis of Genomic and Phenomic Data Reveals Context-Dependent Function of A20 (*TNFAIP3*) in Renal Cell Carcinoma

**DOI:** 10.3390/cancers18111775

**Published:** 2026-05-28

**Authors:** Nour Abu Jayab, Burcu Yener, Reem Sami Alhamidi, Mansi Bhavsar, Alaa Muayad Altaie, Muna Abdalla Alhammadi, Vidya Bijosh Mohan, Marwa Khamis Almazrouei, Lina Sahnoon, Rola Abujabal, Basel Al-Ramadi, Riyad Bendardaf, Iman M. Talaat, Rifat Hamoudi

**Affiliations:** 1 Research Institute of Medical and Health Sciences, University of Sharjah, Sharjah P.O. Box 27272, United Arab Emirates; u21103284@sharjah.ac.ae (N.A.J.); bilce@sharjah.ac.ae (B.Y.); ralhamidi@sharjah.ac.ae (R.S.A.); mbhavsar@sharjah.ac.ae (M.B.); alaa.abed@sharjah.ac.ae (A.M.A.); u21107591@sharjah.ac.ae (M.A.A.); vmohan@sharjah.ac.ae (V.B.M.); marwa.almazrouei@sharjah.ac.ae (M.K.A.); u22105744@sharjah.ac.ae (L.S.); u21105877@sharjah.ac.ae (R.A.); italaat@sharjah.ac.ae (I.M.T.); 2 Department of Clinical Sciences, College of Medicine, University of Sharjah, Sharjah P.O. Box 27272, United Arab Emirates; 3 Center of Excellence for Cancer Research, Research Institute of Medical and Health Sciences, University of Sharjah, Sharjah P.O. Box 27272, United Arab Emirates; riyad.bendardf@uhs.ae; 4 Center of Excellence for Precision Medicine, Research Institute of Medical and Health Sciences, University of Sharjah, Sharjah P.O. Box 27272, United Arab Emirates; 5 Institute for Nutritional Medicine, University of Lübeck, 23562 Lübeck, Germany; 6 Department of Medical Microbiology and Immunology, College of Medicine and Health Sciences, United Arab Emirates University, Al Ain P.O. Box 15551, United Arab Emirates; 7 Oncology Unit, University Hospital Sharjah, Sharjah P.O. Box 72772, United Arab Emirates; 8 Pathology Department, Faculty of Medicine, Alexandria University, Alexandria 21131, Egypt; 9 BIMAI-Lab, Biomedically Informed Artificial Intelligence Laboratory, University of Sharjah, Sharjah P.O. Box 27272, United Arab Emirates; 10 ASPIRE Precision Medicine Research Institute Abu Dhabi, University of Sharjah, Sharjah P.O. Box 27272, United Arab Emirates; 11 Division of Surgery and Interventional Science, University College London, London WC1E 6BT, UK

**Keywords:** renal cell carcinoma, A20, clear cell renal cell carcinoma, NF-κB pathway, integrated analysis

## Abstract

Background: A20, encoded by tumor necrosis factor alpha-induced protein 3 (*TNFAIP3*), is a key negative regulator of NF-κB signaling with context-dependent functions in cancer. Its role in renal cell carcinoma (RCC), particularly clear-cell RCC (ccRCC), remains incompletely defined. Methods: Public GEO transcriptomic data from 23 controls and 32 ccRCC samples were analyzed using gene set enrichment analysis (GSEA). A20-overexpressing HEK293 and 786-O cells were assessed by functional assays and transcriptomic profiling. Eight ccRCC FFPE samples were profiled to compare A20-associated transcriptional patterns with cell-line data. Targeted DNA sequencing was performed in 11 ccRCC and 8 papillary RCC samples, and whole-exome sequencing was conducted in A20-overexpressing 786-O cells. Key genes were further evaluated using Kaplan-Meier survival analysis in 530 ccRCC patients and TCGA-KIRC data comprising 533 primary tumors and 72 normal samples. Results: GEO analysis showed significant *TNFAIP3*/A20 upregulation in ccRCC (*p* = 3.96 × 10^−5^) and enrichment of NF-κB-related gene sets (*p* = 0.01). A20 overexpression produced distinct phenotypes in HEK293 and 786-O cells. In HEK293 cells, A20 increased *BIK* and *ARHGAP6* expression and was associated with increased apoptosis and reduced wound closure. In 786-O cells, A20 suppressed *ARHGAP6* and *APAF1* and was associated with increased proliferation, enhanced wound closure, and reduced apoptosis relative to EV controls. A20-high ccRCC samples showed enrichment of NF-κB, TGF-β, DNA repair, mTOR/metabolic, hypoxia, and proteasome-related pathways. Genomic analyses identified alterations in NF-κB-related genes, including *CARD10* and *IRAK1.* Conclusions: A20/*TNFAIP3* may exert cell-context-dependent effects in RCC and is associated with tumor-relevant transcriptional and genomic alterations requiring further validation.

## Simple Summary

A20 (*TNFAIP3*), a key regulator of NF-κB signaling, has a poorly defined role in renal cell carcinoma (RCC). This study investigates its role mainly in clear-cell RCC (ccRCC) using integrated in silico, transcriptomic, functional, and genomic analyses. We found that A20 is significantly upregulated in ccRCC and associated with NF-κB pathway activation. Functional assays revealed a context-dependent role of A20: in transformed human embryonic kidney-derived cell line, HEK293 cells, A20 promoted apoptosis and reduced migration, whereas in 786-O ccRCC cells, it enhanced proliferation, migration, and survival while suppressing apoptosis. Transcriptomic profiling showed that A20 regulates distinct gene networks, including suppression of *ARHGAP6* and *APAF1* in cancer cells. Patient data confirmed that A20-high tumors exhibit enrichment of oncogenic pathways, including tumor growth factor-β (TGF-β) and DNA repair. Additionally, genomic analyses, including targeted sequencing across RCC subtypes, identified tumor-specific variants. These findings demonstrate that A20 shifts from a tumor-suppressive to a tumor-promoting role in RCC.

## 1. Introduction

A20 is one of the main regulators of the NF-κB pathway, acting through several mechanisms to restrain pathway activation [1]. Many studies have highlighted the critical role of the NF-κB pathway in cancer; however, the focus has involved the prominent members within its three canonical layers: the input layer (e.g., toll-like receptor 5 (TLR5)), the signal transduction layer (e.g., IκBα, p65, and IκK), and the output layer, including anti-apoptotic genes such as *BCL2* and various interleukins [2]. However, beyond these core signaling components, NF-κB activity is tightly controlled by regulatory checkpoints that ensure signal termination and homeostasis. Among these, A20 (Tumor necrosis factor alpha-induced apoptosis 3 (*TNFAIP3*)) functions as a pivotal negative feedback regulator, integrating and fine-tuning NF-κB signaling across multiple levels. A20 is a ubiquitin-modifying enzyme that preserves cellular haemostasis and regulates vital biological processes, including immune response, apoptosis, and inflammation. The unique structure of A20, which is composed of an N-terminal ubiquitin-interacting domain and a C-terminal zinc finger domain, enables A20 to both remove activating ubiquitin chains and catalyze ubiquitin conjugation, thereby tightly controlling NF-κB signal amplitude and duration [3].

A20, encoded by *TNFAIP3*, was initially identified as a tumor necrosis factor-α (TNFα)-inducible gene, linking its critical role to inflammatory signaling and cell death regulation [4]. At the molecular level, A20 regulates the NF-κB pathway through several mechanisms, including binding to TRAF2 and Ubc13, interfering with TRAF6 activation, and promoting ubiquitination and degradation of RIP1. In addition, A20 constrains the activation of the NF-κB essential modulator (NEMO) by linear ubiquitin chain assembly complex (LUBAC) through competing with NEMO for ubiquitination. Through these mechanisms, A20 functions as a critical gatekeeper that fine-tunes the NF-kB activation in immune and inflammatory contexts. Moreover, A20 possesses a conserved binding site for the RelB-p52 complex; hence, it is expressed after NF-κB activation as a negative feedback to limit the NF-κB pathway and maintain cellular haemostasis [5].

The bidirectional, context-dependent role of A20 has been reported across multiple cancer types; however, the molecular mechanisms underlying this duality remain unclear. The role of A20 is most characterized in hematological malignancies, where it is frequently mutated or deleted. A20 loss or dysregulation in lymphomas and myelomas has been shown to drive constitutive activation of the NF-κB pathway, thereby promoting tumor progression and therapeutic resistance [6,7]. In contrast, the role of A20 in solid tumors has been comparatively less explored. Interestingly, A20 has been reported to support tumor-promoting programs in colorectal, breast, and gastric cancers, whereas a tumor-suppressive function has been reported in hepatocellular carcinoma [8], underscoring the strong context dependence of A20 activity across cancer types.

In the renal context, in both diabetic and chronic kidney diseases, A20 is induced as a result of the inflammatory milieu and the activated NF-κB pathway. Under these conditions, A20 is upregulated as part of an endogenous protective response, restraining NF-κB signaling and limiting excessive inflammatory damage. However, in late-stage chronic kidney disease, A20 activation appears insufficient to counterbalance ongoing inflammation and progressive fibrotic remodeling, ultimately failing to restore tissue homeostasis [9].

In cancer, NF-κB-driven A20 is frequently induced during early stages of tumorigenesis as a consequence of uncontrolled NF-κB activation. At this stage, A20 functions as a protective feedback regulator, limiting excessive NF-κB-mediated pro-survival signaling. However, as tumor progression is accompanied by disruption of key cellular checkpoints, the regulatory function of A20 may become altered. In advanced cancer stages, this dysregulation enables A20 to shift toward an anti-apoptotic and tumor-supportive role, contributing to cancer cell survival and disease progression [10].

The role of A20 in renal cell carcinoma (RCC) remains unaddressed. However, in silico analyses of RCC patient datasets show significantly elevated A20 expression in tumor tissues compared with healthy subjects. Moreover, these studies revealed increased expression of NF-κB-associated components, including RelA and IκBα, suggesting sustained NF-κB pathway activation in RCC [2]. Furthermore, A20 expression exhibits great heterogeneity in RCC patients, where higher A20 expression correlates with poor survival, more aggressive and treatment-resistant renal cancer. In line with its established role in cell death regulation, A20 has been implicated in securing renal cells, including tubular and glomerular cells, from TNF-α-induced apoptosis and in limiting immune cell recruitment, hence potentially contributing to immune evasion in the renal tumor microenvironment [11].

As the A20 molecular mechanism in RCC has not yet been studied, the signaling axes and downstream genes mediating its function in RCC remain to be characterized. Given the central role of the NF-κB pathway in regulating key biological processes, including inflammation, metabolism, protein synthesis, and cell migration, and its extensive crosstalk with other oncogenic pathways [12], dissecting A20-mediated NF-κB regulation in RCC is of particular interest.

In this study, we investigated the context-dependent molecular functions of A20 by comparing its effects in the clear cell RCC (ccRCC) in vitro model with those observed in a non-cancerous cell line. In parallel, we analyzed transcriptomic expression data from publicly available ccRCC patient cohorts to assess A20-associated expression patterns in clinical samples. Finally, we performed in vivo differential gene expression analyses with a specific focus on the NF-KB signaling components, including A20 and its related regulatory targets, to elucidate pathways linked to RCC aggressiveness.

## 2. Materials and Methods

**Figure 1:**
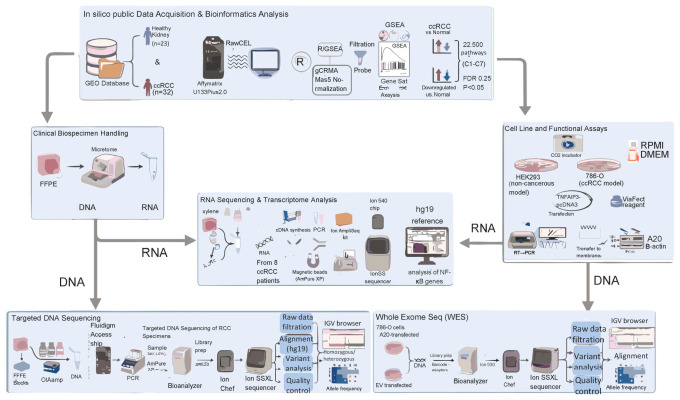
Flowchart outlining the research methodology used. The stepwise flowchart of this study demonstrates the analysis initiated from the GEO repository, comprising publicly accessible microarray datasets of RCC subtypes & normal kidney tissues. MAS5 and gcRMA normalization was performed on these datasets, and gene set enrichment analysis (GSEA) was used to identify pathways with differential activity. After the in silico evaluation, the transformed embryonic kidney cell line (HEK293) and the ccRCC-derived 786-O cell line were seeded, transfected with the TNFAIP3-pcDNA3 construct, and subjected to downstream molecular and functional assays. Furthermore, we performed targeted transcriptome sequencing of ccRCC tissue samples to assess pathway-specific changes in gene expression. Concurrently, we performed whole-exome sequencing and targeted DNA sequencing of genomic DNA extracted from formalin-fixed paraffin-embedded (FFPE) samples of ccRCC and PRCC patients to identify genetic alterations in NF-κB genes. Finally, A20-transfected 786-O cells were assessed by whole-exome sequencing (WES), and the data were analyzed and aligned to hg19. The figure was generated using FigureLabs (https://www.figurelabs.ai) (accessed on 26 February 2026)).

A schematic overview of the research methodology is shown in Figure 1.

### 2.1. In Silico Transcriptomic Analysis of Publicly Available Data Sets for Renal Cell Carcinoma

To highlight the key signaling pathways and significantly differentially expressed genes (DEGs) across RCC subtypes, publicly available transcriptomic datasets were retrieved from the Gene Expression Omnibus (GEO) (https://www.ncbi.nlm.nih.gov/geo/ (accessed on 25 January 2024)). A dataset containing gene expression profiles from multiple RCC subtypes and healthy renal tissues was selected. To minimize platform-related bias, only datasets generated with the Affymetrix Human Genome U133 Plus 2.0 microarray platform (GPL96) were included. The final dataset was derived from the GSE15641 cohort and comprised 23 control and 32 ccRCC cases. A total of 55 Raw CEL Affymetrix files were downloaded and processed for gene set enrichment analysis (GSEA) to identify dysregulated pathways across normal and ccRCC samples.

Each microarray Affymetrix features more than 54,000 probes; hence, the raw CEL files from a total of 55 normal and RCC patients underwent normalization by an in-house R script as previously described [13]. In brief, the Affymetrix Microarray Suite 5 (MAS5) and GeneChip Robust Multiarray Averaging (gcRMA) packages in R v.4.3.2 were employed to normalize the data and subtract background noise. Moreover, invariant probes were excluded from the transcript list, and non-specific filtering was applied to derive a standard set of variant probes. Filtering with default parameters was performed using an R script. Probes with a coefficient of variation (CV) of 10–100% and a MAS5 value > 50 in gcRMA were created and intersected to identify a set of common variant probes.

### 2.2. Cell Culture

This study employed two human renal cell lines: HEK293, an adenovirus type 5-transformed immortalized human embryonic kidney-derived cell line, used as a non-RCC renal cell comparator, and 786-O, utilized as a ccRCC model. HEK293 cells (AddexBio, catalog no. T0011001, San Diego, CA, USA) were maintained in Dulbecco’s Modified Eagle Medium (DMEM)-high glucose (Sigma Aldrich, St. Louis, MO, USA), while 786-O cells (AddexBio, catalog no. C0036002, San Diego, CA, USA) were maintained in Roswell Park Memorial Institute (RPMI) 1640 medium (Sigma Aldrich, St. Louis, MO, USA). Both media were supplemented with 10% fetal bovine serum (Sigma-Aldrich, St. Louis, MO, USA) and 1% Penicillin/Streptomycin (Sigma-Aldrich) at 37 °C in a 5% CO_2_ incubator.

### 2.3. Transfection of HEK293 and 786-O Cells with TNFAIP3

The overexpression of *TNFAIP3* was achieved by transient transfection of cells with TNFAIP3 cloned into the pcDNA3 expression vector. Cells were seeded at a density of 1.5 × 10^5^ per well in 6-well plates 24 h prior to transfection. Both cell lines were transfected with 2 μg of *TNFAIP3* plasmid DNA using ViaFect transfection reagent (Thermo Fisher Scientific, Waltham, MA, USA), following the manufacturer’s protocol. Untreated cells served as a control, and cells treated with ViaFect transfection reagent alone served as mock-transfected controls. Transfection efficiency and *TNFAIP3* overexpression were checked at 24 h post-transfection by measuring *TNFAIP3* messenger RNA (mRNA) levels and A20 protein levels using qRT-PCR and Western blot analysis, respectively.

### 2.4. RNA Extraction

Following 24 h of transfection, cells were collected by trypsinization and pelleted. The RNA was extracted using the RNeasy kit (Qiagen, Hilden, Germany), following the manufacturer’s protocol.

The extracted RNA was further treated with the TURBO DNAase-free TM Kit (Invitrogen, Carlsbad, CA, USA) to ensure complete removal of genomic DNA.

### 2.5. Quantitative Reverse Transcriptase-PCR (qRT-PCR)

Complementary DNA (cDNA) was synthesized from RNA using a High-Capacity cDNA Reverse Transcription Kit (Applied Biosystems, Waltham, MA, USA), following the manufacturer’s guidelines. The cDNA was used to assess gene expression using Maxima SYBR Green/ROX qPCR MasterMix (2×) (Thermo Fisher Scientific) on a QuantStudio 3 (Applied Biosystems, Waltham, MA, USA). The expression of *TNFAIP3* was measured and normalized to 18S using the primer sequences listed in Table 1.

The delta Ct was calculated relative to the internal 18S Ct, and the fold change was measured using the comparative method (2^−∆∆Ct^).

### 2.6. Protein Extraction and Western Blot

At the end of the transfection experiment, cells were pelleted, and protein was extracted using M-PER Mammalian Protein Extraction Reagent (Thermo Fisher Scientific). Also, protease inhibitor cocktails (Sigma Aldrich, St. Louis, MO, USA) and Dithiothreitol (DTT) (Sigma Aldrich, St. Louis, MO, USA) were added to the M-PER reagent. Samples were loaded at 25 μg/well, separated by 10% Sodium dodecyl sulfate-polyacrylamide gel electrophoresis (SDS-PAGE) (Sigma Aldrich, St. Louis, MO, USA), and transferred to a nitrocellulose membrane (Thermo Fisher Scientific). The membrane was blocked with 5% non-fat milk and incubated with Rabbit monoclonal antibody to A20/TNFAIP3 (ab92324, Abcam, Cambridge, UK) at a 1:1000 dilution. Moreover, B-actin antibody at a 1:1000 dilution was used as a housekeeping protein.

### 2.7. Functional Assays

For functional assays, cells were analyzed 48 h post-transfection to allow sufficient time for downstream phenotypic effects of A20 overexpression to manifest, whereas molecular expression analyses (qRT-PCR and Western blotting) were performed at 24 h post-transfection to capture early transcriptional and protein-level changes.

#### 2.7.1. Apoptosis Assay

HEK293 and 786-O cells were seeded in 6-well plates at a density of 2 × 10^5^ cells per well and either left untreated as controls or transfected with empty vector (EV) or A20 expression plasmid. Culture media were then collected and kept on ice. Cells were washed twice with ice-cold phosphate-buffered saline (PBS) (Sigma Aldrich, St. Louis, MO, USA), then incubated with 500 µL of 1× trypsin per well for detachment. Cells were centrifuged at 2000 rpm for 5 min at 4 °C and washed twice with PBS to remove residual media. Apoptosis was assessed using the Annexin V-Fluorescein isothiocyanate (FITC) Apoptosis Detection Kit (Abcam, ab14085) according to the manufacturer’s instructions. Briefly, a master staining solution containing Annexin V-FITC, propidium iodide (PI), and binding buffer was prepared based on the number of samples. Each cell pellet was resuspended in 200 µL of the staining mixture, gently vortexed, and incubated for 20 min at room temperature in the dark. Stained cells were analyzed using a CytoFLEX flow cytometer (Beckman Colter, Brea, CA, USA), and data were analyzed using FlowJo software v10.10.0.

#### 2.7.2. Cell Viability Assay (Trypan Blue Exclusion)

The effect of A20 on cell viability was assessed using the trypan blue exclusion assay. HEK293 and 786-O cells were seeded at a density of 1 × 10^5^ cells per well in 12-well plates. Cells were left untreated as controls or transfected with an EV or an A20 expression plasmid. After 48 h, cells were harvested by trypsinization, and the resulting cell suspensions were stained with 0.4% trypan blue solution. Viable and non-viable cells were counted using a hemocytometer under an inverted microscope. Cell viability was calculated using the following Equation (1):

Cell viability (%) = (number of viable cells/total number of cells) × 100(1)

#### 2.7.3. Edu Cell Proliferation Assay

Cell proliferation was assessed using a 5-ethynyl-2′-deoxyuridine (EdU) incorporation assay (EdU proliferation assay kit; cat. no. ab219801; Abcam, Cambridge, UK). HEK293 and 786-O cells were seeded in 6-well plates at a density of 2 × 10^5^ cells per well and either left untransfected as controls or transfected with EV or A20 expression plasmid. Before collection, cells were treated with 10 µM Edu for 3 h, then processed according to the manufacturer’s protocol. In brief, EdU-treated cells were permeabilized with 1× permeabilization buffer for 20 min at room temperature. Following washing, samples were incubated with a freshly prepared reaction cocktail containing copper sulfate (CuSO_4_, EdU proliferation assay kit), EdU additive solution, and iFluor™ 488 azide (EdU proliferation assay kit) for 30 min at room temperature, protected from light. EdU incorporation was quantified using a CytoFLEX flow cytometer (Beckman Coulter, Brea, CA, USA), and data were analysed using FlowJo software.

#### 2.7.4. Wound-Healing Assays

HEK293 and 786-O cells were seeded and transfected at 80–90% confluency with either an EV or an A20 expression plasmid, while untreated cells served as controls. Twenty-four hours post transfection, a straight scratch wound was generated in each well using a sterile 10 µL pipette tip. Detached cells were removed by washing with 1× PBS, and fresh complete medium was added. Phase-contrast images were captured from three predefined regions along each wound at the indicated time points. As 786-O, a RCC cell line with aggressive migratory behaviour, exhibited near-complete wound closure by 24 h, images of 786-O cells were acquired at 0, 10, and 20 h using a 10× objective lens. In contrast, for HEK293 cells, images were captured at 0, 24, and 48 h using a 4× objective lens. To ensure consistent imaging of the same wound regions over time, reference marks were drawn on the bottom of the plate with a fine marker. Images were acquired using an Olympus DP74 camera (Evident, Tokyo, Japan) mounted on a BX43 microscope (Olympus Life Sciences, Tokyo, Japan).

### 2.8. Cell Lines RNA Sequencing

RNA from EV-transfected and A20-transfected HEK293 and 786-O was subjected to transcriptome profiling using the Ion AmpliSeq™ Transcriptome Human Gene Expression assay on the Ion S5 system (Thermo Fisher Scientific, Waltham, MA, USA) as described previously [14]. Briefly, Turbo DNase treated RNA samples were reverse transcribed into cDNA using the SuperScript VILO cDNA Synthesis Kit (Thermo Fisher Scientific). After the library preparation using the Ion AmpliSeq Transcriptome Human Gene Expression Kit (Thermo Fisher Scientific, Waltham, MA, USA), the library was digested with FuPa reagent (Thermo Fisher Scientific, Waltham, MA, USA) and ligated to adapters. Subsequently, the RNA-seq library was purified using Agencourt AMPure XP Beads (Beckman Coulter, Indianapolis, IN, USA) and quantified using an Ion Library TaqMan™ Quantitation Kit (Applied Biosystems, Thermo Fisher Scientific, Waltham, MA, USA). Finally, the samples were diluted to approximately 100 pM and pooled for sequencing on the Ion S5 XL Semiconductor sequencer using an Ion 540 Chip (Life Technologies Corporation, Carlsbad, CA, USA) prepared on a fully automated Ion Chef System (Thermo Fisher Scientific, Waltham, MA, USA). The RNA-seq data was analyzed using Ion Torrent Software Suite version 5.4 (Thermo Fisher Scientific, Waltham, MA, USA), and the alignment was performed using the Torrent Mapping Alignment Program (TMAP) in reference to the hg19 (GRCh37) assembly. Although hg38 (GRCh38) is a more recent assembly, hg19 was selected for alignment to maintain compatibility with the Ion AmpliSeq Transcriptome panel design, existing analytical pipelines, and downstream annotation resources optimized for hg19, ensuring consistency with prior datasets and established variant/gene models.

### 2.9. Clinical Samples and FFPE-Based Transcriptomic Analysis

#### 2.9.1. Clinical FFPE Renal Biopsies

This retrospective study included formalin-fixed paraffin-embedded (FFPE) renal tissue specimens obtained from the Faculty of Medicine, Alexandria University. Ethical approval for the study was granted by the University of Alexandria, Egypt (IRB No.: 0306337), and the University of Sharjah (REC-23-12-08-01-PG). FFPE specimens were collected from patients diagnosed primarily with RCC who underwent either partial or radical nephrectomy. For the purposes of the current study, 8 ccRCC renal FFPE samples were selected for transcriptomic analyses (Table 2). FFPE blocks were stored at room temperature till further processing and downstream molecular analyses.

#### 2.9.2. RNA Extraction from FFPE

Total RNA was extracted from selected FFPE renal tissue samples for RNA sequencing using the RecoverAll™ Total Nucleic Acid Isolation Kit (Invitrogen, Waltham, MA, USA) according to the manufacturer’s protocol. In brief, 3 μm thick curls were cut from FFPE tissue blocks. The curls were deparaffinized with xylene, followed by two washes with absolute ethanol. After the sample pellets were thoroughly dried, they were digested with protease. Lastly, nucleic acid isolation and subsequent washing steps were performed based on the kit’s instructions. Following RNA extraction, samples were treated with TURBO DNA-free™ kit (Invitrogen, Waltham, MA, USA) to remove genomic DNA prior to downstream applications.

#### 2.9.3. RNA Sequencing of ccRCC FFPE

A total of 8 ccRCC RNA samples were subjected to transcriptomic analysis using the Ion AmpliSeq™ Transcriptome Human Gene Expression assay on the Ion S5 system (ThermoFisher Scientific, Waltham, MA, USA) as described in Section 2.10.

For downstream transcriptomic analysis, ccRCC samples were stratified into A20-high and A20-low groups based on *TNFAIP3* expression. Grouping was performed using a median-centered approach across the 8 samples, with a median *TNFAIP3* expression of 2. Samples with expression values greater than 2 were classified as A20-high, whereas samples with values below 2 were classified as A20-low. Differential gene expression analysis followed by absGSEA was then conducted to identify transcriptional programs associated with A20 status.

### 2.10. Pathway Analysis and Differential Gene Expression in RCC

The enriched cellular pathways from in silico data analysis, cell-line transcriptomic analysis, and tissue transcriptomic analysis were identified using GSEA on the mapped gene list. Each RCC subtype was compared to the normal tissues. An absolute GSEA was performed on the expression data, using approximately 20,500 annotated pathways from seven gene set collections (C5–C7) in the Broad Institute’s Molecular Signatures Database (MSigDB; https://www.gsea-msigdb.org (accessed on 25 January 2024)). Significantly enriched pathways in RCC subtypes were identified using thresholds of FDR < 0.25 and *p* < 0.05 as previously described [15]. These pathways were further analyzed to identify DEGs and their occurrence frequency across pathways using an R script as previously described [16]. Additionally, using statistical methods, a 95th-percentile cutoff was applied to derive gene frequencies across enriched pathways. DESeq2 analysis and a nominal *p*-value < 0.05 were used to identify DEGs for cell- and tissue-level transcriptomic analyses.

### 2.11. Whole Exome Sequencing (WES)

To define the genomic background underlying A20-driven phenotypic and transcriptomic rewiring, we performed whole-exome sequencing (WES) on EV- and A20-transfected 786-O cells and annotated variants using ClinVar [17].

#### 2.11.1. DNA Library Preparation and Sequencing

Genomic DNA extracted from A20-transfected and EV-transfected 786-O cells was processed for whole-exome sequencing using the Ion AmpliSeq Exome RDY Kit (Thermo Fisher Scientific). Briefly, DNA samples were mixed with Ion AmpliSeq HiFi Mix and distributed into the Ion AmpliSeq Exome RDY plate for amplification according to the manufacturer’s protocol. Following amplification, wells were pooled and treated with the FuPa reagent (Thermo Fisher Scientific) for partial digestion. Ion Xpress barcode adapters (Thermo Fisher Scientific) were subsequently ligated, and libraries were purified using Agencourt AMPure XP beads (Beckman Coulter), washed, and eluted in nuclease-free water. Purified libraries were then diluted and quantified by quantitative polymerase chain reaction (qPCR) using the Ion Library TaqMan Quantification Kit (Thermo Fisher Scientific). Final libraries were diluted to approximately 100 pM and sequenced on an Ion 540 chip using the Ion GeneStudio S5 System (Thermo Fisher Scientific).

#### 2.11.2. WES Data Analysis and Variant Filtering

Raw variant calls were generated for A20-overexpressing and two EV-transfected 786-O cell control samples, including genomic locus, reference, and observed alternate alleles, genotype, and functional annotation, using the hg19 (GRCh37) reference genome. Variants were filtered to retain only high-confidence calls within the targeted sequencing renal panel that passed platform-specific quality criteria from the Ion Reporter “Gene lab exome workflow” with default settings.

Variant datasets derived from EV-transfected control samples were merged to generate a non-redundant control variant set, and variants were matched across datasets using a locus- and allele-specific key. Variants detected in the A20-overexpressing sample but absent from both EV control datasets were classified as A20-associated sample-specific variants and retained for downstream analysis.

Variants were further restricted to exonic regions, with a minimum read coverage of >100× and an allele frequency between 5% and 99%. Candidate variants were then visually inspected in Integrative Genomics Viewer (IGV) to confirm read support, strand balance, and alignment quality following concise idiosyncratic gapped alignment report (CIGAR) patterns and to exclude sequencing or alignment artifacts.

### 2.12. Targeted DNA Sequencing

#### 2.12.1. DNA Extraction from FFPE Samples

Genomic DNA was extracted from five control, 11 ccRCC, and 9 papillary RCC (PRCC) FFPE samples (clinical information for all samples is provided in Supplementary Materials Table S1) using the QiaAmp DNA mini kit (Qiagen, Hilden, Germany) according to the manufacturer’s protocol. Non-cancerous kidney samples (inflammatory kidney disease samples) were included to distinguish RCC-associated genetic alterations from non-specific mutations. FFPE-derived PRCC samples were included exclusively for DNA-based analyses to support subtype-level genomic comparisons. Transcriptomic profiling and expression-based stratification were intentionally limited to ccRCC samples to ensure biological consistency.

Briefly, five to ten FFPE sections, each 3 μm thick, were cut from each tissue block and subjected to deparaffinization. Following deparaffinization, tissue pellets were washed with a gradient of alcohol concentrations. The samples were dried completely and digested with 20 μL proteinase K and 180 μL buffer ATL at 56 °C for 2–3 h. Then, 200 μL of Buffer AL and 200 μL 100% ethanol were added to each sample, and lysates were loaded onto QIAamp spin columns. The columns were washed with washing buffer, and DNA was eluted in 30 μL nuclease-free water.

#### 2.12.2. Targeted Next-Generation Sequencing of NF-κB Pathway Genes

Targeted next-generation sequencing (NGS) was performed to identify common variants across multiple genes involved in the NF-κB signaling pathway. DNA extracted from FFPE samples was quantified using NanodropTM (Thermo Fisher Scientific, Waltham, MA, USA). Targeted sequencing was conducted using a Fluidigm Access Array system, as previously described [18]. 42 primers were designed to cover common variants in the NF-κB signaling pathway (Supplementary Materials Table S2). The primers were linked to Fluidigm-specific tag sequences: CS1: ACACTGACGACATGGTTCTACA for the forward primer and CS2: TACGGTAGCAGAGACTTGGTCT for the reverse primer.

Briefly, around 60 ng of DNA from each sample was amplified with 10 µM of added tagged primer using the Fast Start High Fidelity master mix (Roche, Basel, Switzerland). The amplified products were then purified using ExoSAP-IT (Invitrogen, Waltham, MA, USA) according to the manufacturer’s protocol. The purified amplicons were then amplified using tagged primers on the 48.48 Access Array integrated fluidic circuit (IFC), using Fast Start High Fidelity Master Mix (Roche, Basel, Switzerland). After the multiplex PCR, products were extracted from the Fluidigm 48.48 IFC (Fluidigm Europe B.V., Amsterdam, The Netherlands). The harvested products from each sample inlet was diluted 100-fold using nuclease-free water. Then, the diluted product was attached to the access array barcode library. The amplicon library was purified using AMPure XP beads (Beckman Coulter, Brea, CA, USA). After that, for quantification, A High Sensitivity DNA assay kit on a BioAnalyzer (Agilent, Santa Clara, CA, USA) was used. The libraries were diluted to 100 pM and sequenced on an Ion S5 XL Semiconductor sequencer using an Ion 520 Chip (Life Technologies Corporation, Carlsbad, CA, USA) prepared on a fully automated Ion Chef System (Thermo Fisher Scientific, Waltham, MA, USA).

#### 2.12.3. DNA Sequencing Data Analysis

The DNA-targeted sequencing data were processed using an in-house bioinformatics pipeline that involved raw data filtering, alignment to the hg19 (GRCh37) reference genome, and comprehensive quality control, as previously described [13]. Binary alignment map (BAM) files were analyzed using Integrative Genomics Viewer (IGV) version 2.15.4 (Broad Institute, Cambridge, MA, USA) [18,19,20]. A minimum of 12 reads was considered a cut-off for each sample. The criteria also included that the mutation is only detected in tumor samples and is absent from all non-cancerous control kidney samples. Based on the percentage of the mutated allele, individuals were classified as homozygous or heterozygous. Samples with a rate of mutated allele less than 20% are labeled as homozygous wild type. Heterozygous alleles were identified as percentage greater than or equal to 20% and less than 80%. Homozygous mutants have an allele frequency of 80% or higher. Mutation frequency was calculated using the following Equation (2):

(2) $$\mathrm{Mutation Frequency}=\frac{\mathrm{number of patients with the specific mutation}}{\mathrm{Total number of Read}-\mathrm{positive patients}}\times 100$$

### 2.13. Validation of Genes Related to A20 Overexpression in ccRCC

Survival analysis was performed using the Kaplan-Meier Plotter database (https://kmplot.com/analysis/ (accessed on 29 April 2026)) to generate the overall survival (OS) curves for some of the key genes identified after GSEA analysis of A20-low and A20-high samples. The *ARHGAP6*, *CARD10*, and *IRAK1* genes were included in the analysis. In addition, the university of Alabama at Birmingham Cancer data analysis portal (UALCAN) (http://ualcan.path.uab.edu (accessed on 29 April 2026)) bioinformatics tool was used to assess the expression of *TNFAIP3*, *IRAK1*, and *CARD10* in the kidney renal clear cell carcinoma (KIRC) The Cancer Genome Atlas (TCGA) dataset, comprising 72 normal samples and 533 primary tumor samples.

### 2.14. Statistical Analysis

RNAseq data of the cell line and FFPE tissue were analyzed using the DESeq2 package (v1.38.3) in R (v4.3.2) to identify DEGs [21]. Genes showing an absolute log2 fold change <−1.5 and >1.5 and a *p*-value below 0.05 were considered significantly altered. *p*-values were corrected for multiple testing using the Benjamini–Hochberg false discovery rate (FDR) method [15]. Statistical analyses for in vitro experiments were performed using GraphPad Prism (v10.0) and are presented as mean ± standard deviation (SD) from three independent biological replicates, each performed in technical triplicate. Comparisons between two groups were conducted using unpaired Student’s *t*-tests (or Welch’s *t*-test when variances were unequal), while comparisons among multiple groups were performed using one-way ANOVA followed by Tukey’s post hoc test. These parametric tests were applied to control vitro datasets. For boxplots, the first and third quartiles defined the lower and upper hinges, the central line represented the median, and whiskers extended to data points within 1.5 times the interquartile range (IQR).

A schematic overview of the experimental design and integrative analytical workflow employed in this study is presented in Figure 1.

## 3. Results

### 3.1. GSEA of Publicly Available ccRCC Datasets Reveals NF-κB Pathway Activation and Increased A20 Expression

**Figure 2:**
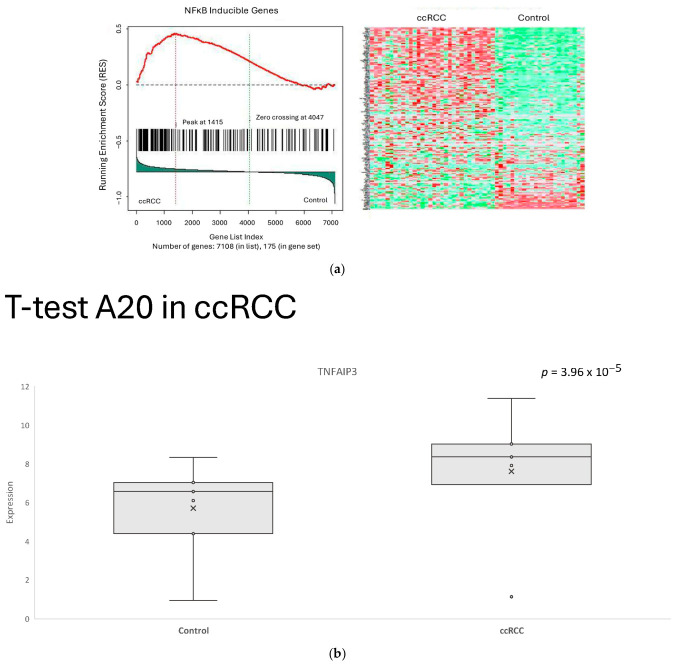
Transcriptomic enrichment analysis identifies NF-κB activation and elevated A20 expression in ccRCC patients. (**a**) Gene Set Enrichment Analysis (GSEA) performed on publicly available microarray data from the Gene Expression Omnibus (GEO; GSE15641) revealed significantly enriched pathways in 32 ccRCC samples compared with 23 normal kidney tissues, including strong enrichment of the NF-κB pathway (*p* = 0.018). (**b**) A20 (TNFAIP3) expression levels in ccRCC versus healthy controls, showing significantly higher A20 expression in tumor samples (*p* = 3.96 × 10^−5^). Heatmaps show expression profiles of genes in the representative pathways. Red indicates high expression, while green indicates low expression.

Absolute gene set enrichment analysis (absGSEA) was performed on publicly available transcriptomic data as an exploratory in silico approach to identify pathways dysregulated in ccRCC relative to normal renal tissue. Comparative analyses between 23 Normal and 32 ccRCC samples revealed significant enrichment of multiple cancer-associated pathways. Significant pathways were identified based on *p* value < 0.05 and FDR < 0.25. Notably, the NF-κB pathway emerged as one of the top-enriched pathways with a *p* value of 0.018, indicating sustained activation of inflammatory and pro-survival signaling in ccRCC (Figure 2a). Moreover, consistent with enhanced NF-κB pathway activity, A20 expression was significantly increased in ccRCC samples compared with normal renal tissues (*p* value = 3.96 × 10^−5^) (Figure 2b). Together, these findings indicate that NF-κB-associated signaling and its key regulatory component, *TNFAIP3*/A20, are altered in ccRCC. This analysis was used as an exploratory reference and was not intended for direct quantitative comparison with RNA-sequencing datasets. The complete list of significantly enriched pathways from the C2 (curated gene sets), C5 (Gene Ontology), and C7 (immunologic signatures) collections, as well as the DEGs identified from the comparison between healthy controls and ccRCC patients, is provided in Supplementary Materials Tables S3A and S4A, respectively.

While FFPE-derived patient samples revealed clinically relevant A20-associated transcriptional programs, they do not permit direct assessment of causality. To determine whether A20 overexpression is sufficient to drive the transcriptional changes observed in A20-high ccRCC tumors, we next employed two cell lines with distinct biological and genetic backgrounds. HEK293 cells were used as a transformed renal model derived from human embryonic kidney tissue, whereas 786-O cells served as a ccRCC model that exhibits the key phenotypic and genetic mutational backgrounds of the disease.

### 3.2. Overexpression of A20 in HEK293 and 786-O Cell Lines Induces Robust Increases at Both mRNA and Protein Levels

**Figure 3:**
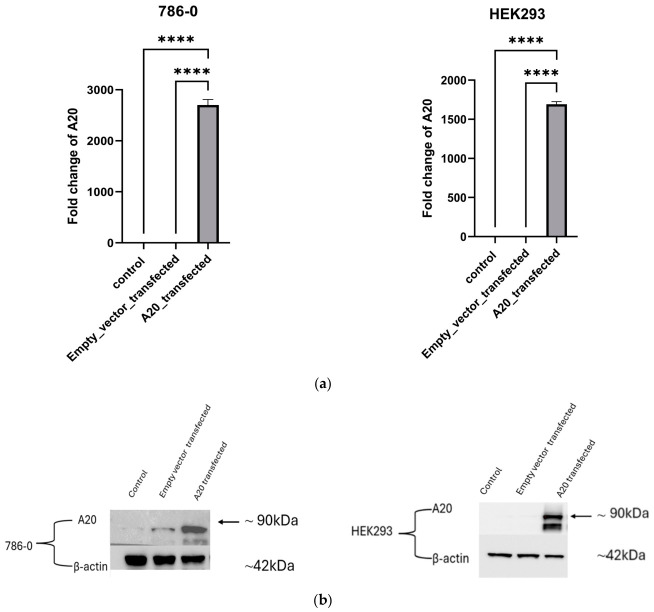
Validation of A20 overexpression in HEK293 and 786-O cells at mRNA and protein levels. (**a**) RT-qPCR quantification of A20 expression following 24 h transient transfection with the A20-pcDNA3 construct or EV, showing 1692-fold and 2700-fold increases in HEK293 and 786-0 cells, respectively. (**b**) Western blot confirming successful A20 protein overexpression relative to empty-vector control. Data are presented as mean ± SD from three independent experiments (**** *p* < 0.0001).

Transient transfection of both HEK293 and 786-0 cell lines with either an empty pcDNA3 vector or a *TNFAIP3* expression construct resulted in a marked increase in A20 expression at both the transcript and protein levels. Quantitative RT-qPCR analysis revealed a substantial upregulation of *TNFAIP3* mRNA, with an average increase of approximately 1692-fold in HEK293 cells and 2700-fold in 786-0 cells relative to empty-vector and control (Figure 3a).

Consistent with the transcript-level data, Western blot analysis confirmed strong upregulation of A20 protein in both cell lines compared with control and pcDNA3 empty-vector (EV)-transfected cells (Figure 3b). These results confirm efficient A20 overexpression in both HEK293 and 786-O cellular models.

### 3.3. A20 Overexpression Differentially Modulates Apoptosis in HEK293 and 786-O Renal Cells

**Figure 4:**
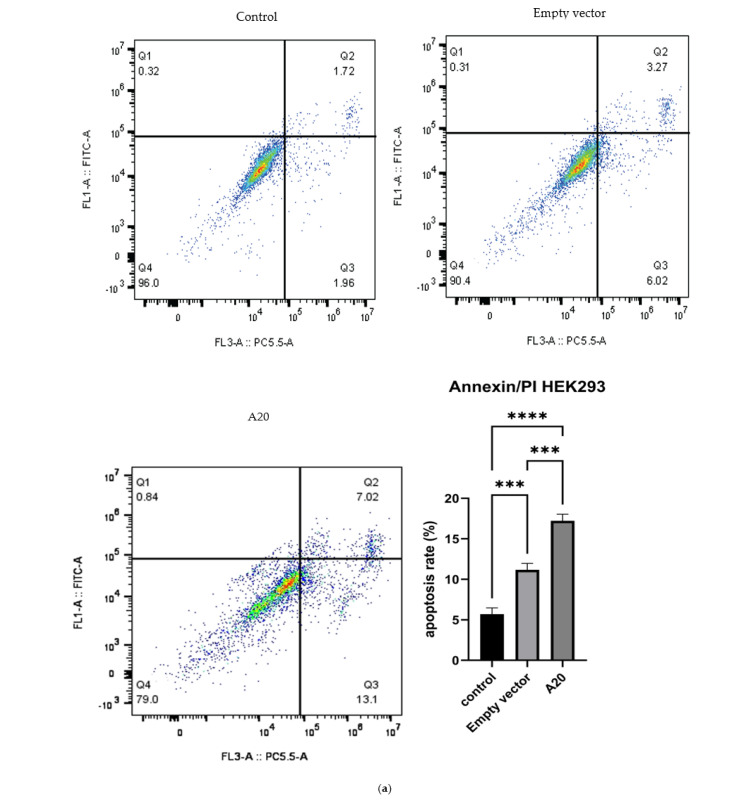

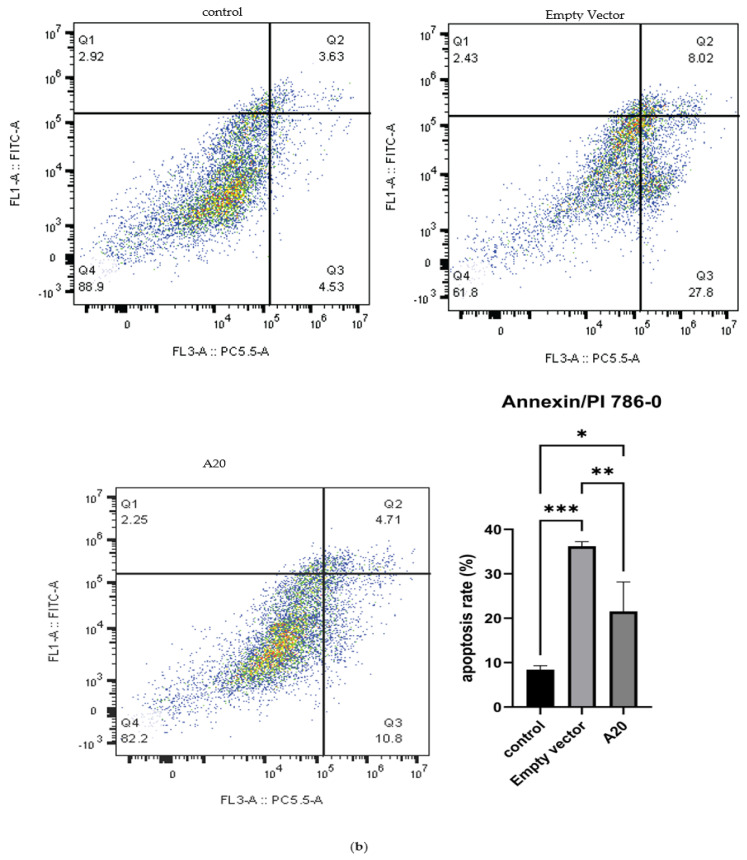

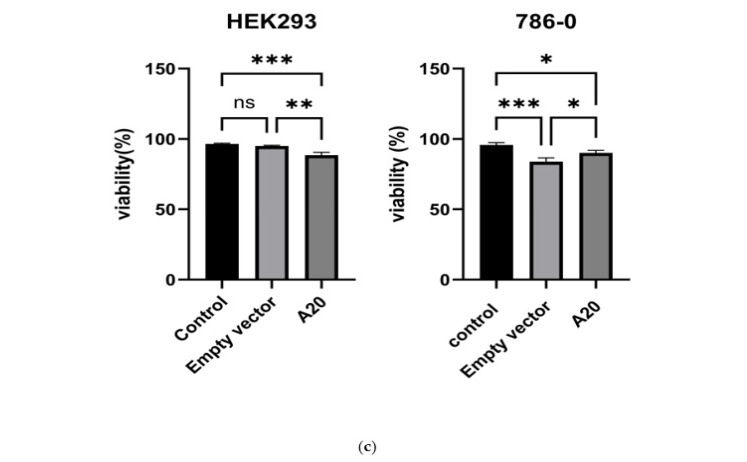
A20 differentially regulates apoptosis in HEK293 and 786-O renal cells. (**a**) Flow-cytometry quantification of Annexin-V/PI staining showing significantly increased apoptosis in A20-overexpressing HEK293 cells (*p* < 0.0001). (**b**) Corresponding apoptosis levels in 786-O cells, where A20 reduces apoptosis relative to the empty vector, but remains higher than that of the untreated control. Dot color in panels (**a**,**b**) indicate event density: blue = low density, green = medium density, and yellow/red = highest density. (**c**) Trypan blue viability assay demonstrating reduced viability in A20-overexpressing HEK293 cells but enhanced viability in 786-0 cells. Data are presented as mean ± SD from three independent experiments. Statistical analysis was performed using one-way ANOVA followed by Tukey’s post hoc test for multiple comparisons. * *p* < 0.05, ** *p* < 0.01, *** *p* < 0.001, **** *p* < 0.0001; ns, not significant.

A20 overexpression resulted in distinct apoptotic responses in each renal cell line. Data derived from three independent biological replicates demonstrated that A20 overexpression significantly increased total apoptotic cell population in HEK293 cells compared with control conditions (*p* < 0.0001) (Figure 4a). This increase was evident across combined early and late apoptotic fractions, indicating enhanced apoptotic susceptibility in HEK293 cells.

In contrast, 786-O cells overexpressing A20 exhibited higher apoptotic rates than untreated control cells, but significantly lower apoptosis compared with EV-transfected cells, suggesting an attenuation of apoptosis in the ccRCC context (Figure 4b).

These results were further supported by trypan blue exclusion-based cell viability analysis. Both cell lines were transfected with A20 for 48 h HEK293 cells displayed a significant reduction in viable cell numbers compared with both control and EV-transfected cells, whereas A20 overexpression significantly increased cell viability in 786-O renal cancer cells (Figure 4c), consistent with a survival-promoting effect in the malignant setting.

### 3.4. A20 Differentially Regulates Proliferation and Wound Closure in HEK293 and 786-O Renal Cells

**Figure 5:**
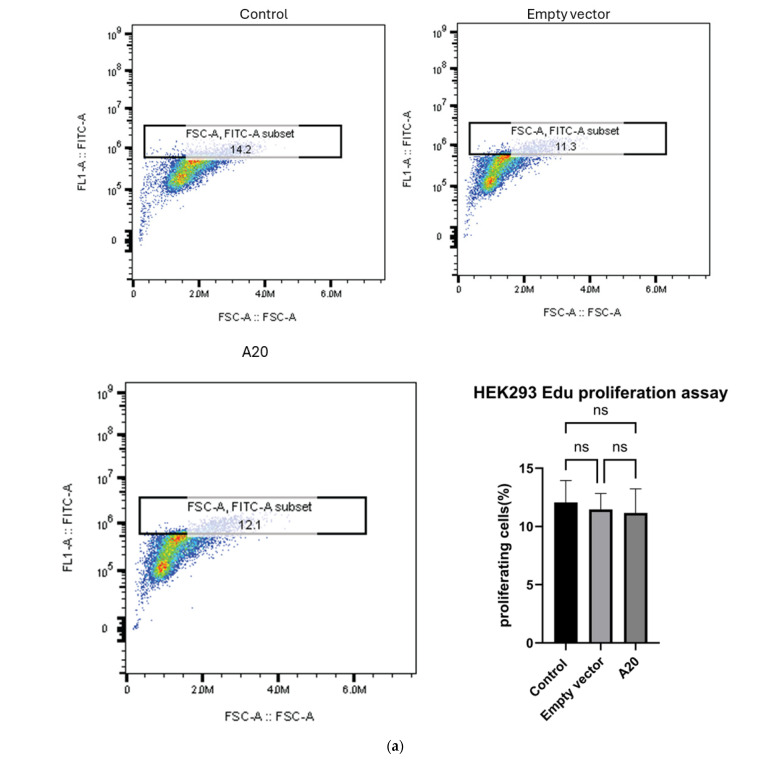

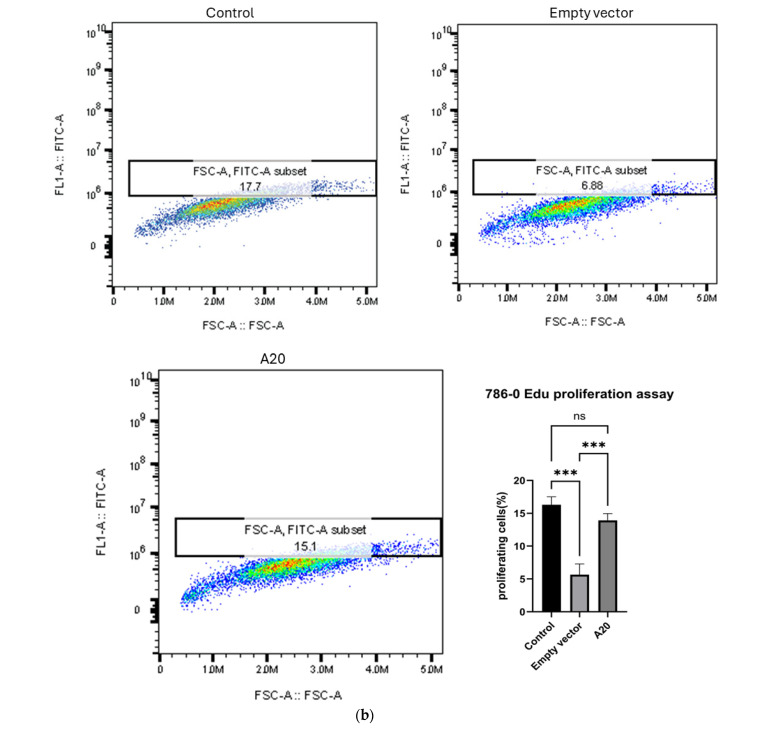

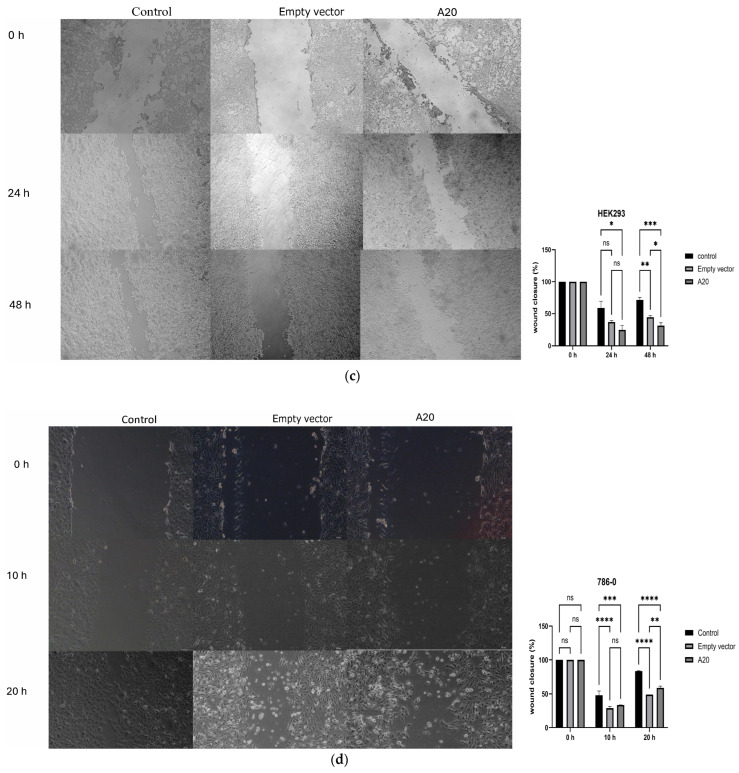
A20 modulates proliferation and wound closure in a cell-type-dependent manner. (**a**) EdU proliferation assay showed no significant change in proliferation in A20-overexpressing HEK293 cells compared with empty-vector controls. (**b**) An Edu proliferation assay of 786-O cells overexpressing A20 showed a significant increase in proliferation. Data are presented as mean ± SD from three independent experiments. Statistical analysis was performed using one-way ANOVA followed by Tukey’s post hoc test for multiple comparisons. *** *p* < 0.001; ns, not significant. (**c**) Wound healing assay of HEK293 cells measured at 0, 24, and 48 h, showing markedly reduced wound closure in A20-overexpressing cells compared with empty-vector, indicating impaired migration. (**d**) Wound-healing assay of 786-O cells measured at 0, 10, and 20 h, demonstrating accelerated wound closure and enhanced migratory capacity in A20-overexpressing cells relative to empty-vector. Data are presented as mean ± SD from three independent experiments. Statistical analysis was performed using two-way ANOVA followed by multiple-comparisons testing. * *p* < 0.05, ** *p* < 0.01, *** *p* < 0.001, **** *p* < 0.0001; ns, not significant.

Functional assays were next performed to determine whether A20 modulates cell proliferation and migratory capacity in a context-dependent manner. EdU incorporation analysis revealed that A20 overexpression did not significantly alter proliferation in HEK293 cells with comparable EdU-positive fractions observed across control, EV, and A20-transfected conditions (Figure 5a). In contrast, A20 overexpression significantly increased proliferation in 786-O cells compared with EV-transfected cells, based on data obtained from three independent biological replicates (Figure 5b). Notably, while EV transfection markedly suppressed proliferation, A20 restored proliferative capacity to levels comparable to untreated controls.

Wound closure was subsequently assessed using wound-healing assays. In HEK293 cells, notably, A20 overexpression resulted in significantly delayed wound closure compared with EV-transfected cells at both 24 and 48 h (Figure 5c). Conversely, 786-O cells overexpressing A20 exhibited significantly accelerated wound closure, particularly at later time points, relative to EV-transfected cells (Figure 5d).

Collectively, these findings demonstrate that A20 exerts divergent effects on proliferation and wound closure in HEK293 versus 786-O renal cells, reinforcing its context-dependent functional role in renal cell biology.

### 3.5. A20 Overexpression Induces Distinct Transcriptomic Profile in HEK293 and 786-O Cells

To assess how A20 overexpression reshapes global gene expression patterns, whole-transcriptome sequencing was performed in HEK293 and 786-O cells following transfection with either an A20 expression vector or the EV. Transcriptome analysis confirmed a clear separation between A20-overexpressing cells and their corresponding control samples in both HEK293 and 786-O (Supplementary Materials Table S3B,C), indicating robust A20-driven transcriptional reprogramming.

**Figure 6:**
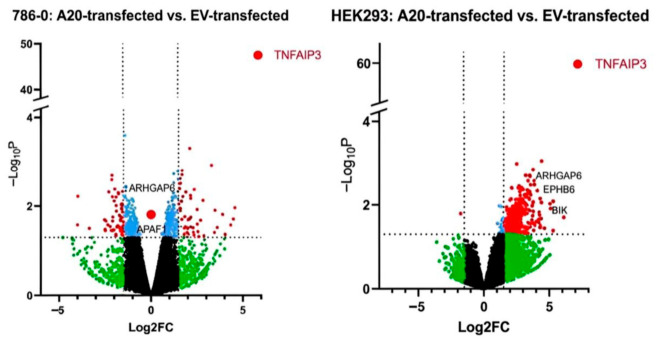
Volcano plots of differentially expressed genes. Genes that are expressed significantly higher in either EV- or A20-transfected cell line, based on <−1.5 and >1.5 log2-fold change, *p* < 0.05 are highlighted by red dots, genes with statistical significance only (*p* < 0.05), but not meeting the log2-fold change threshold are shown in blue, *p* > 0.05 are highlighted by green dots (Log2FC NS), and unchanged transcripts are demarcated as black (NS). NS, Not Significant.

After normalization and data filtering, 663 genes were identified as significantly differentially expressed in A20-overexpressing HEK293 cells, while 518 differentially expressed genes (DEGs) were detected in A20-overexpressing 786-0 cells, each compared with their respective control conditions (Figure 6). Complete DEG lists for both cell lines are provided in the Supplementary Materials Table S4B,C. The most strongly upregulated and downregulated genes, defined by log2-fold changes greater than 1.5 or less than −1.5 and *p* value < 0.05, are indicated in the volcano plots, illustrating distinct transcriptional responses to A20 overexpression in the two cellular contexts.

Leading-edge analysis revealed that genes involved in apoptosis, proliferation, and DNA repair machinery were dysregulated following A20 overexpression. These genes were derived from the most significantly enriched gene sets and differed between the two cell models. In HEK293 cells, prominent leading-edge genes comprised *BIK, ARHGAP6*, and *EPHB6*, whereas in 786-O cells, leading-edge genes comprised *APAF1*, *DAPK1*, *ERCC1*, *INO80C*, *BCL2L12*, and *ARHGAP6*, reflecting distinct A20-associated transcriptional programs in HEK293 versus 786-O cells.

### 3.6. A20 Differentially Regulates Cytoskeletal and Migration-Associated Transcriptional Programs in HEK293 and 786-O Cells, Highlighting ARHGAP6

**Figure 7:**
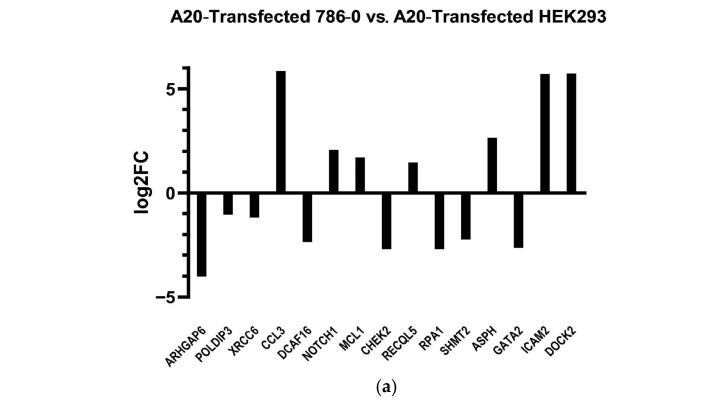

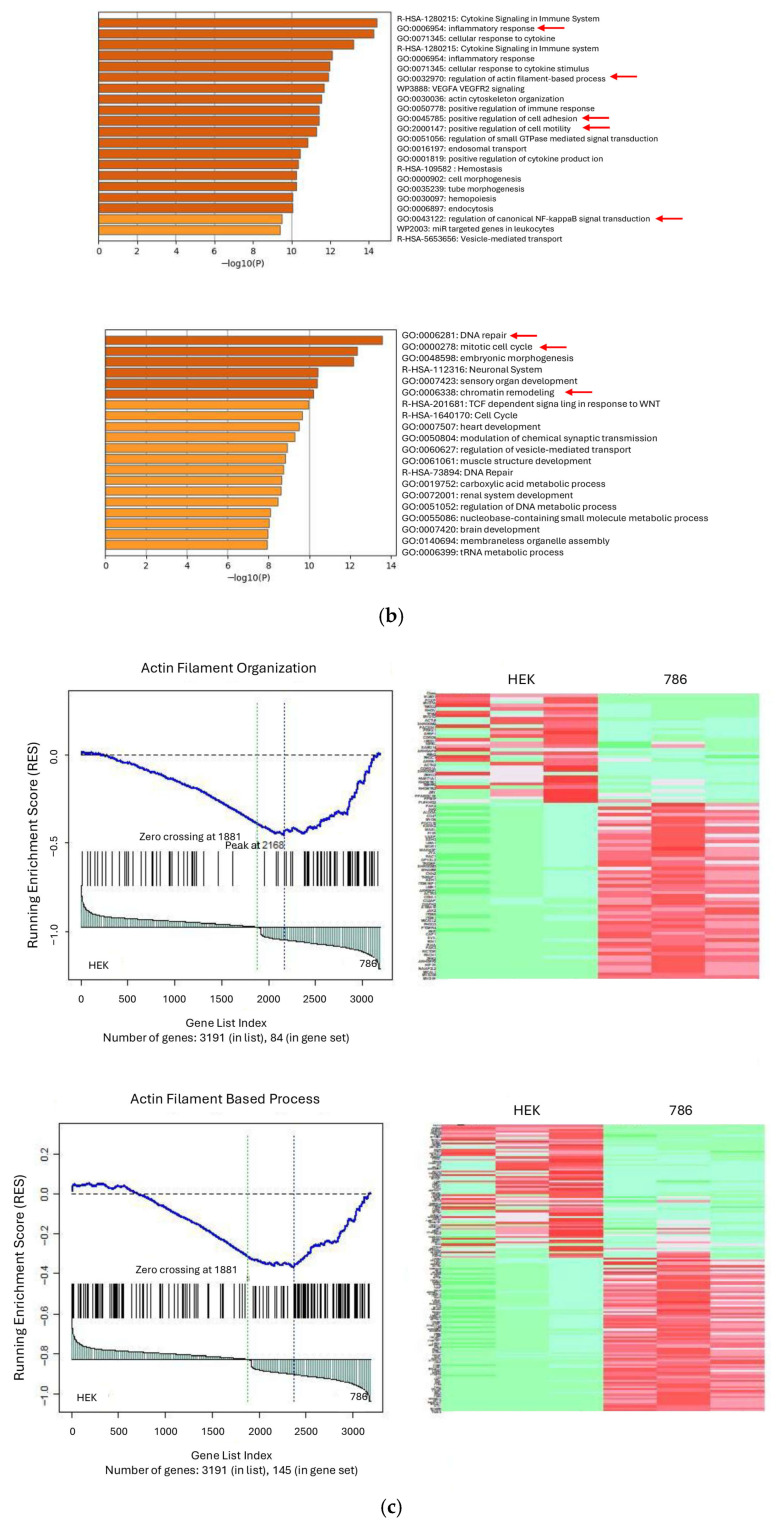
Key A20-responsive genes and pathways differ between HEK293 and 786-O renal cells. (**a**) Histogram of top activated genes in A20-overexpressing 786-O vs. A20-overexpressing HEK293 after filtration and normalization from EV-transfected cells. (**b**) Significant enrichment pathways based on the top significant genes in A20-transfected 786-O vs. A20-transfected HEK293 cell line (*p* < 0.001). (**c**) Heatmap visualization of genes in both gene sets revealed consistent expression differences between the two groups, with enrichment of actin-related genes in A20-transfected 786-O cells compared with A20-transfected HEK293 cells. The red arrows indicate the common enriched pathway between A20-high ccRCC patients and A20-transfected 786-O cells. Heatmaps show expression profiles of genes in the representative pathways. Red indicates high expression, while green indicates low expression.

To directly compare A20-dependent transcriptional effects between HEK293 and 786-O cells, gene expression profiles of A20-transfected HEK293 cells were compared with those of A20-transfected 786-O cells, following filtering against EV controls to minimize baseline cell-line effects. This analysis identified distinct sets of genes selectively regulated by A20 in each cellular context (Figure 7a). The DEGs lists resulting from A20 overexpression in 786-O vs. HEK293 are listed in the Supplementary Materials Table S4D.

In 786-O RCC cells, A20 overexpression reshaped gene expression in a manner that reinforces multiple cancer hallmarks, including enhanced cell migration, survival, and DNA repair-related processes, thereby promoting a tumor-supportive phenotype. In contrast, in HEK293 transformed embryonic kidney cells, A20-associated gene expression patterns were enriched for pathways related to cellular homeostasis, apoptosis, and regulated migration, reflecting a more physiological and regulatory response rather than a tumor-promoting one (Figure 7a,b).

Notably, Rho GTPase activating protein 6 (*ARHGAP6*) emerged as a recurrent A20-responsive gene in both cellular models, although with opposing expression patterns. In HEK293 cells, A20 overexpression was associated with increased *ARHGAP6* expression, whereas in 786-O cells, *ARHGAP6* expression was reduced relative to controls (Figure 7a). Given ARHGAP6’s established role in regulating Rho GTPase signaling and cytoskeletal dynamics, this divergent regulation suggests a context-dependent involvement of *ARHGAP6* in A20-mediated transcriptional programs. Moreover. The list of significantly enriched pathways that were derived from absGSEA in A20-overexpressed 786-O vs. A20-overexpressed HEK293 is provided in Supplementary Materials Table S3D.

GSEA further revealed significant enrichment of GO: Actin Filament Organization and GO: Actin Filament-Based Process pathways in A20-overexpressing 786-O cells compared with A20-overexpressing HEK293 cells. Enrichment plots showed a coordinated shift of actin-associated genes toward 786-O expression profile, indicating differential cytoskeletal regulation between the two cell types. Heatmap analysis further confirmed differential expression of genes involved in actin dynamics between the two cell types. Consistently, heatmap analysis confirmed distinct expression patterns of genes involved in actin dynamics across HEK293 and 786-O cells (Figure 7c). Collectively, these findings suggest that A20 overexpression differentially modulates cytoskeletal and migration-related transcriptional programs in HEK293 versus 786-O cells, with *ARHGAP6* representing a shared but directionally distinct A20-responsive gene across cellular contexts.

### 3.7. Transcriptional Profiling of ccRCC Patient Samples Based on A20 Expression Levels

Cell line-based models are inherently homogeneous and two-dimensional culture systems, but do not fully reflect the architecture of a real tumor or its surrounding microenvironment. To evaluate whether the A20-associated transcriptional programs observed in vitro are reflected in patient samples, transcriptomic profiling was performed on FFPE tissue specimens from 8 ccRCC patients exhibiting variable A20 expression.

Patients were stratified into A20-high and A20-low groups based on RNA-seq expression values using a predefined cutoff of 15. Samples with expression values below this threshold were classified as A20-low (*n* = 4), whereas samples above the threshold were classified as A20-high (*n* = 4). Consistent with cell-line transcriptomic analysis, differential gene expression revealed a unique expression profile for each patient group. The full list of DEGs is provided in Supplementary Materials Table S4E.

**Figure 8:**
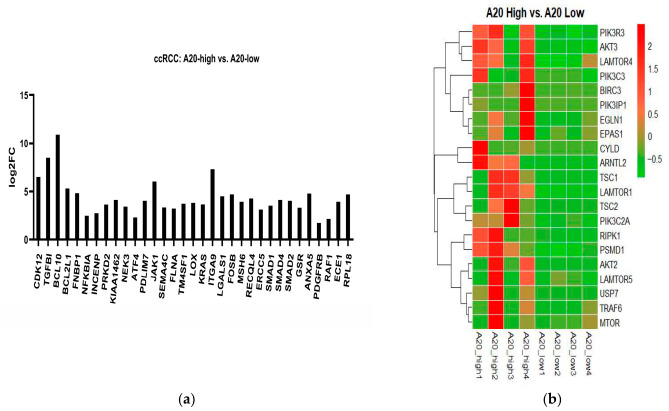
Selected leading-edge genes in A20-high vs. A20-low ccRCC patients. (**a**) Histogram showing the top activated genes identified by comparing A20-high FFPE ccRCC samples (*n* = 4) with A20-low ccRCC samples (*n* = 4). (**b**) Heat map of the selected leading-edge genes related to metabolic reprogramming and hypoxia response across the same group sample.

Interestingly, more than 3000 genes were upregulated in A20-high ccRCC patients compared with A20-low patients. These genes drive major cancer hallmarks, including invasion, immune escape, and hypoxia-driven metabolic reprogramming (Figure 8a,b).

**Figure 9:**
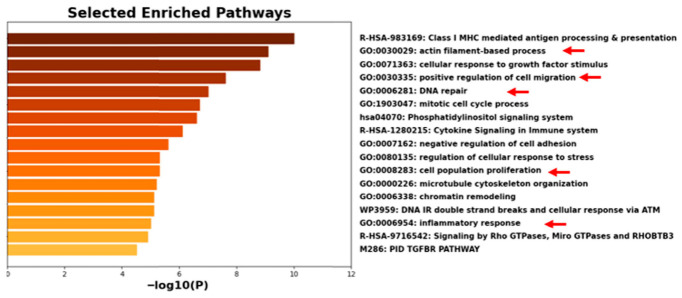
Significant enrichment pathways of the most upregulated genes in A20-high vs. A20-low ccRCC. Color intensity indicates the statistical significance of pathway enrichment, with darker brown indicating greater significance. Red arrows indicate pathways commonly enriched in A20-high ccRCC patients and A20-transfected 786-O cells.

Moreover, the annotation of the most significantly upregulated genes in A20-high ccRCC tumors showed enrichment of pathways related to metabolic processes, hypoxia response, DNA repair, proteasome pathways, cellular stress response, growth, chromatin remodeling, and TGF-β signaling (Figure 9 and Supplementary Materials Figure S1A–C). These pathway enrichments indicate broad transcriptional differences between A20-high and A20-low patient groups and highlight signaling programs selectively associated with elevated A20 expression in ccRCC (Supplementary Materials Table S3E).

### 3.8. Whole-Exome Sequencing Reveals A20-Associated Clonal Selection in Genomically Heterogeneous 786-O Cells

Given transcriptomic signals implicating stress adaptation and dysregulation of DNA repair pathways, we performed whole-exome sequencing to identify variants in A20-overexpressing cells compared with EV-transfected 786-O cells. The whole-exome sequencing achieved 95.73% target base coverage, reflecting strong capture efficiency and high overall sequencing quality. Initial variant calling identified 50,368 raw variants in the A20-overexpressing sample and 49,059 variants in the EV control. After restricting the analysis to targeted exonic regions and applying standard quality control filters, 819 variants remained in the A20-overexpressing sample and 793 variants in the control dataset. Comparative analysis revealed 321 shared variants, while 498 variants were detected exclusively in the A20-overexpressing sample.

To focus on high-confidence alterations, further filtering was applied to retain single-nucleotide variants (SNVs), insertions/deletions (INDELs), and multi-nucleotide variants (MNVs) with allele frequencies between 5–99% and a minimum sequencing depth was above 100. This yielded 19 high-confidence variants, including pathogenic and benign or variants of uncertain significance shown in Table 3. Functionally relevant variants were predominantly missense alterations (*n* = 9) affecting cancer- and inflammation-associated genes, while remaining variants were largely synonymous and predicted to be benign (*n* = 10).

Notably, among the variants detected in the A20-overexpressing condition were pathogenic missense mutations in *TP53* and *MR1*, as well as a risk-associated variant in the NF-κB upstream receptor *TLR5*. Consistent with the genomic heterogeneity of RCC cells, these variants likely reflect selective expansion of pre-existing subclonal populations under A20-mediated survival pressure rather than de novo mutational events.

To assess whether genes harboring variants in A20-overexpressing cells participate in coordinated biological pathways rather than representing isolated genomic events, we performed pathway enrichment analysis using Metascape (http://metascape.org, accessed on 20 January 2026), including *TNFAIP3* (*A20*) and all genes identified in the variant dataset. This analysis revealed significant enrichment of interconnected biological processes related to immune signaling, NF-κB regulation, cellular stress responses, senescence, and autophagy in cancer (Supplementary Materials Table S5). Notably, multiple variant-bearing genes, including *TP53*, *TLR5*, Interleukin 1 receptor-associated kinase 1 (*IRAK1*), caspase recruitment domain family member 10 (*CARD10*), Major histocompatibility complex, class I-related (*MR1*), and *DAPK1*, clustered within these A20-centered functional networks. These findings further support a coordinated remodeling of inflammatory and survival signaling in the context of A20 overexpression.

### 3.9. Targeted DNA Sequencing Identifies Novel A20 Variants in ccRCC and PRCC

**Figure 10:**
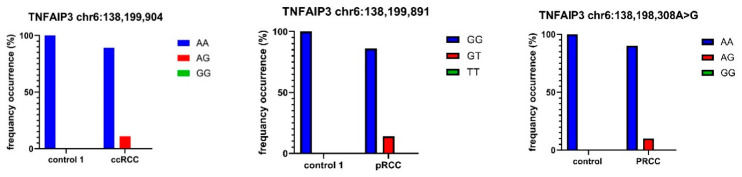
Targeted DNA sequencing detects novel tumor-specific SNVs in A20 and NF-κB pathway genes. Detection of two novel A20 SNVs in RCC patient tumor: chr6:138199904 A>G (ccRCC) and chr6:138199891 G>T (PRCC).

Targeted DNA sequencing identified two novel single-nucleotide variants (SNVs) within the *TNFAIP3* (*A20*) gene that were detected exclusively in tumor samples and were absent from healthy control subjects. The variant chr6:138199904 A>G was identified in 1 of 8 ccRCC patients, whereas chr6:138199891 G>T was detected in 1 out of 6 PRCC patients. Both variants met the predefined quality criteria, including a minimum read depth of ≥10 and visual confirmation using Integrative Genomics Viewer (IGV) (Figure 10). In addition to *A20*, multiple SNVs were detected across other NF-κB-related pathway genes, as summarized in Table 4. Together, these targeted sequencing data further support the presence of tumor-specific genomic alterations involving A20 and NF-κB signaling components across renal cancer subtypes.

### 3.10. Validation of Genes Related to A20 Overexpression

**Figure 11:**
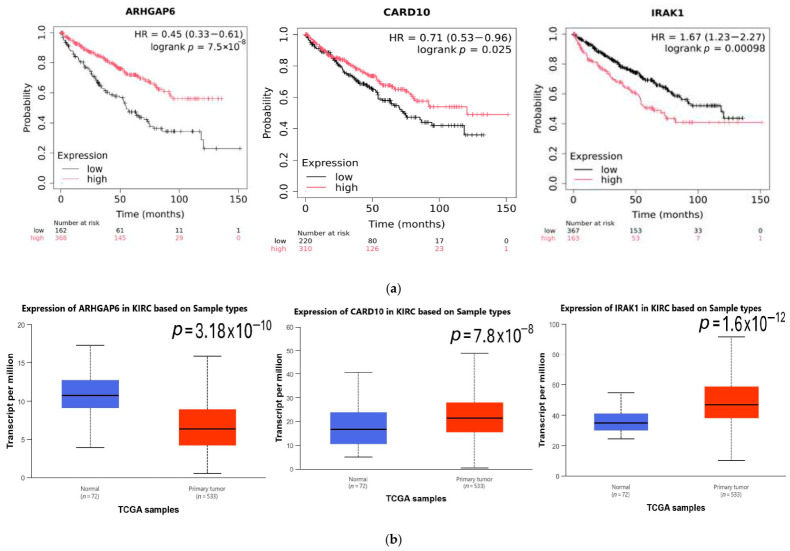
Association of *ARHGAP6*, *CARD10*, and *IRAK1* expression with overall survival and differential expression in ccRCC. (**a**) Kaplan–Meier survival analyses of 530 ccRCC patients stratified by high and low expression of *ARHGAP6*, *CARD10*, and *IRAK1.* Hazard ratios (HR) with 95% confidence intervals and log-rank *p* values are shown for each gene. Survival probabilities are plotted over time (months), with numbers at risk indicated below each panel. (https://kmplot.com (accessed on 4 January 2026)). (**b**) TCGA-KIRC expression analysis of *ARHGAP6*, *CARD10*, and *IRAK1* in 72 normal kidney samples and 533 primary tumor samples using the UALCAN database. Gene expression levels are shown as transcripts per million (TPM). Statistical significance between normal and tumor groups was assessed using an unpaired Student’s *t*-test. (http://ualcan.path.uab.edu, accessed on 29 April 2026).

To validate key genes identified in this study, survival analysis was performed using the Kaplan-Meier Plotter in a cohort of 530 ccRCC patients. *ARHGAP6*, *CARD10*, and *IRAK1* were selected for survival analysis based on their recurrent identification across transcriptomic and genetic analyses, their functional association with A20-regulated pathways, and their involvement in cytoskeletal regulation and NF-κB signaling. Notably, higher expression levels of *ARHGAP6* and *CARD10* were significantly associated with improved overall survival (OS) in ccRCC patients, whereas higher *IRAK1* expression was significantly associated with worse survival (Figure 11a). In addition, TCGA-KIRC expression analysis using the UALCAN database showed that *ARHGAP6* expression was significantly downregulated in 533 primary tumor samples compared with 72 normal kidney samples, whereas *CARD10* and *IRAK1* were significantly upregulated in primary tumor samples (Figure 11b). Together, these findings suggest that *ARHGAP6*, *CARD10*, and *IRAK1* may contribute to ccRCC pathogenesis and indicate that A20-associated effects may involve the coordinated regulation of multiple signaling pathways and protein networks.

## 4. Discussion

RCC remains one of the most challenging urologic malignancies due to its heterogeneity, treatment resistance, and the lack of reliable biomarkers for early detection and patient stratification [22,23]. Despite advances in targeted therapies and immunotherapies, the mechanistic understanding of RCC progression remains incomplete. In this study, we integrated transcriptomic, functional, and genomic approaches to define A20-associated molecular programs in RCC, revealing coordinated alterations centered on NF-κB signaling.

The present study employed a cross-layer framework in which transcriptomic, genomic, and phenotypic data were examined sequentially to investigate the context-dependent role of A20/*TNFAIP3* in RCC. This approach allowed functional observations from cell-line assays to be interpreted alongside transcriptional and genetic alterations observed in patient-derived and public datasets [24].

In this study, the in silico, transcriptomic, and functional analyses were used to identify A20/TNFAIP3-associated pathway-level changes and to generate hypotheses for future mechanistic studies. Accordingly, pathway enrichment results should be interpreted as associative transcriptional patterns rather than direct causal mechanisms.

Notably, our exploratory in silico analysis of publicly available GEO ccRCC datasets revealed enrichment of the NF-κB-related gene set in ccRCC patient samples, accompanied by upregulation of A20 (*TNFAIP3*). These findings suggest that the NF-κB-A20 axis may be altered in ccRCC and support further investigation; however, this dataset was used for exploratory validation and not for direct cross-platform comparison [25].

Consistent with this concept, recent evidence indicates that WDR54 promotes hepatocellular carcinoma progression by enhancing NF-κB signaling via an epigenetic mechanism at the p65 promoter. This supports the broader concept that NF-κB activity in cancer can be regulated by upstream modulators and epigenetic mechanisms. Therefore, although our findings suggest an association between A20/*TNFAIP3* and NF-κB-related transcriptional changes in RCC, future studies are needed to define the precise mechanisms by which A20 modulates NF-κB signaling in this context [26].

Although A20 is a well-established regulator of NF-κB signaling, its role in solid tumors is highly context-dependent, exhibiting tumor-suppressive or oncogenic functions depending on the cellular background [27,28].

Our functional experiments demonstrate that A20 exerts strikingly context-dependent effects in renal cells. In the HEK293 line, A20 overexpression significantly increased apoptosis, attenuated wound closure without altering proliferation, consistent with a pro-apoptotic of A20 [29]. By contrast, in the RCC-derived 786-0 cancer cell line, A20 promoted multiple hallmarks of tumorigenicity, including enhanced cell viability, proliferation, and wound closure, while simultaneously attenuating apoptosis. Although context-dependent roles for A20 have been suggested in other cancer types, our study provides the first direct functional evidence that A20 can switch from a tumor-suppressive to a tumor-promoting role specifically within the RCC setting. These results indicate that A20 may potentiate oncogenic signaling in a setting where canonical inhibitory checkpoints are disrupted [30].

The context-dependent effects of A20 observed between HEK293 and 786-O cells likely reflect fundamental differences in cellular background rather than a uniform A20-driven mechanism. In ccRCC-derived 786-O cells, loss of functional VHL leads to constitutive activation of hypoxia-associated signaling and metabolic rewiring, which can intersect with NF-κB pathway activity and stress adaptation programs. In this setting, A20-mediated modulation of ubiquitin-dependent signaling may reinforce pro-survival and proliferative signaling networks. In contrast, HEK293 cells are a transformed embryonic kidney-derived cell line with wild-type *VHL*; therefore, they do not recapitulate the *VHL*-loss/HIF-activated context of ccRCC [31].

Additional factors that may contribute to this context specificity include differences in TP53 status, basal NF-κB activity, epigenetic landscape, and metabolic state, all of which can influence how A20 regulates downstream signaling. Given that A20 functions as a ubiquitin-editing enzyme acting on multiple signaling intermediates, its net effect is likely shaped by the availability of upstream stimuli and pathway dependencies in a given cellular context. Therefore, the divergent phenotypic responses observed in HEK293 and 786-O cells may reflect differential integration of A20 within pre-existing signaling networks. These considerations highlight the need for future studies to directly dissect the contribution of VHL status, NF-κB signaling dynamics, and other context-defining factors in mediating A20-dependent effects in RCC [32].

Interestingly, transfection with the EV alone was associated with a marked cellular stress response in 786-O cells, reflecting the intrinsic vulnerability of 786-O cells to perturbations in cellular homeostasis. This response may be partly caused by an innate immune response triggered by the exogenous DNA vector or the transfection process. However, the apoptotic response and proliferation were significantly attenuated with A20 overexpression compared with EV, suggesting that A20 may function as a stress-adaptive survival factor in 786-O.

Transcriptomic profiling in both cell lines indicated that A20 overexpression was associated with context-dependent changes in gene expression networks related to apoptosis, proliferation, migration, and DNA repair. In HEK293 cells, A20 overexpression was associated with increased expression of pro-apoptotic and anti-migratory programs. In contrast, in 786-O ccRCC cells, A20 overexpression was associated with reduced expression of tumor-suppressive regulators, including *ARHGAP6*, together with enrichment of transcriptional programs related to invasive signaling, DNA repair, and TGF-β-associated pathway, consistent with aggressive tumor behavior. These findings suggest that A20-associated transcriptional changes may differ between transformed embryonic kidney-derived HEK293 cells and ccRCC cells (786-O). Therefore, these results support an association between A20 expression and altered transcriptional pathway activity, but further functional validation is required to determine whether A20 directly regulates these pathways.

In line with the cell-line transcriptomic findings, whole-transcriptome profiling of FFPE samples from ccRCC patients showed that A20-high ccRCC tissue samples exhibited altered transcriptional programs associated with proliferation metabolism, DNA repair, and TGF-β-related signaling. Specifically, A20-high samples showed enrichment of genes including *KRAS*, *PIK3C3*, *CDK12*, and several components of the TGF-β signaling axis, such as SMAD family members, *TGBI*, *PDGFRB*, *RPS6K1*, and *FBLN5*. Because TGF-β signaling has been implicated in epithelial-mesenchymal transition, matrix remodeling, angiogenesis, and tumor invasiveness in RCC, these findings suggest a potential association between high A20 expression and transcriptional programs linked to more aggressive tumor features [33,34]. Furthermore, key DNA repair regulators, including *MSH6*, *RECQL4*, and *ERCC5*, were differentially expressed, suggesting that A20-high RCC tissues may be associated with transcriptional programs linked to genomic stress responses [35].

However, these observations remain correlative and should not be interpreted as direct evidence that A20 regulates these genes or pathways. Rather, they identify candidate transcriptional programs for hypothesis-generating associated with A20-high tumors that require further validation in a larger independent cohort and functional mechanistic studies.

Consistently, GSEA indicated that A20-high tumors were enriched for pathways related to mitochondrial metabolism, hypoxia response, and proteasome function. These pathway-level associations suggest a potential link between A20 expression and metabolic or stress-adaptive transcriptional programs in ccRCC, consistent with the established hypoxia-driven biology of this disease [36]. Proteasome-related enrichment may reflect increased ubiquitin-proteasome activity, in line with the role of A20/*TNFAIP3* as a ubiquitin-editing regulator of NF-κB signaling. However, these findings are correlative and require functional validation.

The enrichment of mTOR- and metabolism-related pathways in A20-high ccRCC patients is consistent with the metabolic reprogramming nature of ccRCC. Recent pan-RCC analyses further show that RCC heterogeneity may reflect transcriptomic and metabolic states beyond histopathologic classification, including mitochondrial gene signatures that are prognostically relevant. These findings suggest that A20/TNFAIP3-associated transcriptional programs may intersect with mitochondrial function, hypoxia-driven metabolic adaptation, and mTOR-related signaling. Future studies are needed to determine whether A20 contributes to subtype-specific metabolic adaptation or genomic stress responses across RCC subtypes [37].

To define the genomic context accompanying A20-driven transcriptional changes, whole-exome sequencing was performed in A20-overexpressing 786-O cells. Given the short overexpression window (24 h), the detected variants are unlikely to represent de novo mutational events but instead reflect selective expansion of pre-existing subclones within a genomically heterogeneous RCC population. This pattern indicates that A20 functions as a context-dependent survival modulator, reshaping cellular heterogeneity by favoring clones with enhanced stress tolerance, immune resistance, and sustained NF-κB signaling. Notably, pathogenic alterations affecting *TP53* and immune-regulatory genes such as *MR1* and *TLR5* further support this adaptive selection model [38,39,40,41].

In addition to these alterations, whole-exome sequencing identified a *CARD10* missense mutation that is uniquely present in A20-transfected 786-O cells. *CARD10* encodes a scaffolding protein that links receptor-mediated signaling, particularly G-protein-coupled receptor (GPCR) and receptor tyrosine kinase, to downstream NF-κB activation via the CARD10–BCL10–MALT1 (CBM) signalosome. Dysregulation of *CARD10* has been implicated in RCC proliferation and invasion [42]. The emergence of *CARD10* alterations under sustained A20 expression suggests compensatory selection to preserve pro-survival NF-κB output. Importantly, *CARD10* mutations were also detected in RCC patient samples, reinforcing the clinical relevance of this adaptive signaling network.

Taken together, the mutation profile observed in A20-overexpressing 786-O cells, spanning genomic instability (*TP53*), and immune escape (*MR1*, *TLR5*), supports a unified model in which A20 reshapes tumor evolution by promoting the survival of clones adapted to genomic stress and immune pressure. This integrated mechanism aligns closely with prior findings in RCC, in which tumor aggressiveness often reflects the combined effects of immune evasion and genomic deregulation [39,43,44,45].

These observations may reflect early clonal divergence or technical variation rather than definitive biological effects. Further validation will be required to determine whether these differences represent reproducible A20-associated genomic patterns. Future studies incorporating genome-wide analyses across additional cellular models will be required to validate whether A20-associated genomic patterns reflect a cancer-specific adaptive process or a broader consequence of prolonged A20 perturbation.

To further connect the whole-exome sequencing findings with patient data, this study also examined genetic alterations in A20 across ccRCC and PRCC tumor tissues compared with healthy controls. Three tumor-specific SNVs were identified, including two variants affecting exon 6 within the OTU catalytic domain of A20 and one variant located in exon 7 within a zinc-finger ubiquitin-binding domain.

The OTU catalytic domain of A20 is essential for terminating NF-κB signaling through deubiquitinating key mediators, while the zinc-finger domains regulate ubiquitin binding and proteasomal targeting of signaling components. Variants affecting exon 6 and exon 7, therefore, have the potential to disrupt both catalytic and regulatory functions of A20, favoring prolonged NF-κB activation and survival signaling [46]. The presence of tumor-specific variants impacting both OTU and zinc-finger domains in patient samples highlights a convergent pattern of functional A20 disruption, likely neutralizing its homeostatic regulatory role and enabling sustained oncogenic NF-κB signaling in RCC [47,48].

Moreover, targeted DNA sequencing revealed several mutations across NF-κB-related genes, including *CARD10*, *IRAK1*, *TLR2*, and *BCL3* in PRCC patient samples. Importantly, the identified alterations in *IRAK1* and *CARD10* indicate that these mutations are not limited to in vitro A20-driven clonal selection but are also present in clinical tumor tissue. As *IRAK1* and *CARD10* are upstream regulators of canonical NF-κB, their concurrent alteration suggests functional cooperation that may amplify inflammatory and pro-survival signaling in RCC [49].

Although these A20 variants were detected at low frequency, their tumor-specific occurrence reflects RCC heterogeneity rather than a common driver event and is characteristic of regulatory signaling adaptors. Together with the transcriptomic and functional data, these findings support a context-dependent shift of A20 from tumor-suppressive regulation toward oncogenic facilitation [40].

Overall, our integrative analyses demonstrate that A20 drives coordinated functional, transcriptional, and genomic remodeling in RCC, promoting oncogenic phenotypes through NF-κB signaling, inflammatory responses, and altered genome maintenance. Further investigation of A20-associated pathways may open new avenues for improving RCC prognosis and expanding the currently limited biomarker landscape [50].

Moreover, our findings support the concept that RCC is driven in part by chronic inflammatory signaling and sustained activation of pro-survival pathways, particularly NF-κB. Persistent immune dysregulation has been widely implicated in tumor initiation, progression, and immune evasion, and ccRCC tumors are known to arise within an inflammatory microenvironment enriched in cytokines and oxidative stress that amplifies NF-κB activity and promotes tumorigenesis [51].

Our data further support these observations by showing that NF-κB pathway activation is significantly enriched in ccRCC tissues and accompanied by elevated A20 expression, consistent with a persistent inflammatory state underlying tumor biology. Beyond cancer, NF-κB plays a central role in chronic renal inflammatory diseases, including diabetic kidney disease and chronic kidney disease, where sustained activation drives cellular stress and tissue damage [52].

Although A20 is induced as a compensatory anti-inflammatory regulator in these settings, this response frequently becomes dysfunctional in advanced disease, contributing to loss of immune control and a microenvironment permissive to tumor development. These findings highlight the importance of inflammatory context in shaping RCC progression and therapeutic response [53].

Together, our findings emphasize the importance of individualized approaches to RCC diagnosis and treatment based on patient-specific inflammatory and genetic profiles, particularly NF-κB activation and A20 dysregulation. Given the heterogeneity of A20 expression and its association with prognosis and therapeutic resistance, integrating inflammation-related transcriptional biomarkers may improve risk stratification and clinical decision-making. Future studies in larger cohorts will be required to validate these signatures and determine how A20-centered pathways can be leveraged to enhance treatment response and personalized RCC management.

This study has several limitations that should be acknowledged. First, an overexpression-based system may not fully recapitulate endogenous A20 regulation, as it resulted in supraphysiological expression levels. Such high levels of A20 expression may induce non-specific cellular stress responses or transcriptional artifacts and may not accurately reflect physiological signaling dynamics. Therefore, the observed effects should be interpreted as model-dependent or amplified responses rather than precise representations of endogenous A20 function Alternative approaches, such as CRISPR-mediated activation or a titrated A20 expression system, could provide deeper mechanistic insight. In addition, the present study used HEK293 cells as the non-cancerous kidney-derived comparator. However, HEK293 cells are transformed embryonic kidney-derived cells, and therefore do not fully recapitulate the biology of normal renal epithelial cells particularly with respect to NF-κB signaling and apoptotic regulation. Therefore, the observed differences between HEK293 and RCC cells should be interpreted as model-dependent responses rather than direct comparisons of normal versus cancer renal biology. Future validation using primary renal proximal tubular epithelial cells or immortalized non-transformed renal epithelial cells will be required to confirm the physiological relevance of A20 in non-malignant renal contexts. A limitation of the wound healing assay is the absence of mitomycin C; therefore, wound closure may reflect both proliferation and migration. Future studies should include proliferation-controlled wound healing assays and a complementary trans-well migration assay. Also, one limitation of the functional assays is that 786-O cells were sensitive to the transfection procedure, as evidenced by EV-associated cellular stress. Future studies should include mock-transfected controls and alternative transfection approaches, such as stable transfection.

Another limitation is that the WES analysis lacks validation using biological replicates and/or orthogonal sequencing approaches. Without such validation, the detected variants should be interpreted cautiously, as they may include stochastic or technical variation. Also, the non-cancerous comparator samples used for targeted DNA sequencing were obtained from individuals with inflammatory diseases. Due to the practical challenges associated with obtaining kidney tissue biopsies from healthy donors, available non-cancerous kidney samples were used as comparator tissues. Furthermore, the small cohort size is primarily attributable to the lower prevalence of RCC relative to other solid tumors. Therefore, the transcriptomic findings require validation in larger independent cohorts [54,55]. In addition, the integrative transcriptomic analyses included heterogeneous platforms (in silico analysis of GEO microarray data, FFPE tissue, and cells AmpliSeq-based RNA-seq), which may introduce batch effects and limit direct quantitative comparability. To minimize these effects, each dataset was processed and normalized within its own platform-specific framework, and integrative analyses were restricted to directional consistency and pathway-level convergence rather than direct comparison of expression magnitudes. Finally, one limitation of the present study is that the mechanistic basis of A20 function in RCC was not investigated. Although our transcriptomic and pathway enrichment analyses identified associations with NF-κB-related, ubiquitin/proteasome-related, hypoxia response, DNA repair, and TGF-β signaling pathways, these findings remain correlative. Therefore, without direct mechanistic validation, it is not possible to determine whether the observed A20-associated effects are mediated by NF-κB signaling or other pathways are involved. Future studies using co-immunoprecipitation, ubiquitination assays, NF-κB reporter assays, and Electromobility shift assay (EMSA)-based NF-κB DNA-binding analysis.

## 5. Conclusions

This study provides cross-layer evidence suggesting that A20/TNFAIP3 may influence renal cell behavior and ccRCC-associated transcriptional programs in a context-dependent manner. Transcriptomic profiling of A20-transfected HEK293 and 786-O cells showed distinct gene expression patterns associated with apoptosis, proliferation, DNA repair, and cellular stress responses. Among the differentially regulated genes, ARHGAP6 exhibited an opposite expression pattern, increasing in HEK293 cells and decreasing in 786-O cells following A20 overexpression. These changes, together with altered expression of apoptosis-related genes such as BIK and APAF1, may contribute to the divergent apoptotic and proliferative phenotypes observed in the two cell models. In ccRCC tissue-based analyses, A20/TNFAIP3 expression was increased in tumor samples compared with normal renal tissue. Pathway enrichment analyses further suggested that A20-high ccRCC samples are associated with transcriptional programs related to NF-κB signaling, TGF-β signaling, cell-cycle regulation, hypoxia response, proteasome activity, and metabolic remodeling. These findings support a potential role for A20/TNFAIP3 in ccRCC biology and suggest that A20-associated transcriptional changes may intersect with tumor-relevant signaling and stress-adaptive pathways. Overall, our findings indicate that A20/TNFAIP3 may have cell-context-dependent effects in renal models and may be associated with altered signaling programs in ccRCC. However, given the exploratory nature of the transcriptomic and pathway-level analyses, the limited patient cohort size, and the use of an overexpression model, further validation in larger independent cohorts and mechanistic studies using inducible expression and loss-of-function approaches is required to confirm the causal role of A20/TNFAIP3 in RCC progression.

## Figures and Tables

**Table 1:** Forward and reverse qRT-PCR primers sequence used.

Gene	Forward Primer	Reverse Primer
*TNFAIP3*	CATCATTTTGTACCCTTGGTG	GTCTTCAAATCTTCCCCGGTC
*18s*	TGACTCAACACGGGAAACC	TCGCTCCACCAACTAAGAAC

**Table 2:** Clinical and demographic characteristics of ccRCC patients included in the transcriptomic analysis.

Characteristic	N = 8 ^1^
Age	61 (51, 66)
Sex	
Female	3 (37.5%)
Male	5 (62.5%)
Diagnosis	
ccRCC	8 (100%)
Nuclear_grade	
1	1 (12.5%)
2	3 (37.5%)
3	4 (50%)
4	0 (0%)
Capsular_invasion	
Negative	4 (50%)
Positive	4 (50%)
Renal_sinus_invasion	
Negative	8 (100%)
Extent	
Localized	8 (100%)
Ki67_expression	
Negative	4 (50%)
Positive	4 (50%)
Systemic treatment	2 (25%)
Status	
Die of disease	0 (0%)
Totally cured	8 (100%)

^1^ Median (interquartile range (IQR)); n (%).

**Table 3:** Somatic Variants Identified in A20 overexpressed 786-O cells (Compared to Matched EV transfected 786-O cells).

Locus	Genes	Coding	Amino Acid Change	Variant Effect	Frequency %	Coverage	dbSNP	ClinVar
chr22:37898673 C>T	*CARD10*	c.1723G>A	p.Val575Met	missense	22.48	218	rs773495772	Not Reported
chrX:153284993 G>A	*IRAK1*, *MIR718*	c.193C>T	p.Arg65Cys	missense	14.16	219	rs2065766605	Not Reported
chr10:27324581 T>A	*ANKRD26*	c.2798A>T	p.Asn933Ile	missense	15.84	101	rs1410439472	Not Reported
chr11:118392832 G>A	*KMT2A*	c.11864G>A	p.Ser3955Asn	missense	20.37	162	rs782721532	Not Reported
chr1:181018236 A>G	*MR1*	c.116A>G	p.His39Arg	missense	54.82	166	rs2236410	Likely benign
chr1:181018212 G>A	*MR1*	c.92G>A	p.Arg31His	missense	55.15	165	rs41268456	Pathogenic
chr17:7577098 T>A	*TP53*	c.840A>T	p.Arg280Ser	missense	34.58	107	rs1567547687	Pathogenic
chr1:150529990 C>A	*ADAMTSL4*	c.2068C>A	p.Leu690Ile	missense	24.58	118	Not Reported	Not Reported
chr1:223284599 T>C	*TLR5*	c.1775A>G	p.Asn592Ser	missense	59.26	135	rs2072493	risk factor
chr1:67787457 C>T	*IL12RB2*	c.249C>T	p.His83=	synonymous	23.81	105	rs1206626828	Not Reported
chr1:150526406 C>T	*ADAMTSL4*	c.939C>T	p.Gly313=	synonymous	50.24	205	rs6681639	Benign
chr8:118832020 G>A	*EXT1*	c.1431C>T	p.Pro477=	synonymous	15.38	130	rs17439693	Benign
chr9:90321583 C>T	*DAPK1*	c.3597C>T	p.Arg1199=	synonymous	26.71	161	rs3118863	Not Reported
chr13:33628239 G>A	*KL*	c.1155G>A	p.Lys385=	synonymous	30.28	109	rs1871862189	Benign
chr1:150526472 G>A	*ADAMTSL4*	c.1005G>A	p.Pro335=	synonymous	18.41	201	rs759155216	Likely benign
chrX:149680539 C>T	*MAMLD1*	c.2193C>T	p.Cys731=	synonymous	59.44	143	rs113311895	Not Reported
chrX:149680554 C>T	*MAMLD1*	c.2208C>T	p.Ser736=	synonymous	59.29	140	rs2070779	Not Reported
chr19:8645703 C>G	*ADAMTS10*	c.*74G>C	p.?	unknown	15.7	172	rs1348389927	Not Reported
chr17:7460685 A>A	*TNFSF13*	c.-1672G>A	p.?	unknown	99.3	143	rs1128963	Benign

**Locus** is chromosome location and altered nucleotide, **Frequency %** is percentage of sequencing reads at a given genomic position that support the alternate (mutant) allele, **Coverage** is a total read at the locus. In protein-level notation, “=” indicates no amino acid change, while “?” indicates an unknown protein effect.

**Table 4:** Summarizing additional SNVs detected across NF-κB pathway genes.

Allele Type	SNV	Corresponding Gene	Frequency (%)	RCC Subtype	SNV ID
Heterozygous	chr22:37,912,254:C>T	*CARD10*	25% (1/4)	PRCC	rs199764326
Heterozygous	chr22:37,912,263:T>C	*CARD10*	25% (1/4)	PRCC	unreported
Heterozygous	chr22:37,912,165:C>T	*CARD10*	25% (1/4)	PRCC	unreported
Heterozygous	chr18:56,414,933:T>C	*MALT1*	17% (1/6)	PRCC	rs752917364
Homozygous	chr19:45,260,660:C>T	*BCL3*	20% (1/5)	PRCC	unreported
Heterozygous	chrX:153,278,861:C>T	*IRAK1*	20% (1/5)	PRCC	unreported
Heterozygous	chr20:6,758,880:C>T	*BMP2*	25% (1/4)	PRCC	unreported
Heterozygous	chr20:6,758,885:A>G	*BMP2*	25% (1/4)	PRCC	unreported
Homozygous	chr11:32,456,387:T>C	*WT1*	33% (1/3)	PRCC	unreported
Heterozygous	chr4:154,624,260:T>C	*TLR2*	20% (1/5)	PRCC	unreported

**Frequency (%)** is the frequency of patients harboring the mutations.

## Data Availability

The RNA sequencing data generated in this study have been deposited in the sequence read archive (SRA) under SRA Series access number PRJNA1420462 and can be accessed from (https://www.ncbi.nlm.nih.gov/sra/?term=PRJNA1420462, accessed on 9 February 2026), and in GEO under accession number GSE285469 (https://www.ncbi.nlm.nih.gov/geo/query/acc.cgi?acc=GSE285469, accessed on 20 December 2025). All other supporting generated data are either included in the manuscript or available on request from the corresponding author.

## Supplementary Materials

The following supporting information can be downloaded at: https://www.mdpi.com/article/10.3390/cancers18111775/s1, Figure S1: GSEA of enriched pathways in A20-high vs. A20-low ccRCC FFPE samples. (**A**) GSEA of proteasome gene module in A20-high vs. A20-low ccRCC patients. (**B**) GSEA of cellular response to hypoxia in A20-high vs. A20-low ccRCC patients. (**C**) GSEA of aerobic respiration to hypoxia in A20-high vs. A20-low ccRCC patients, Table S1: Demographic and clinicopathological data, Table S2: Primer’s list and sequence of DNA-targeted sequencing, Table S3A: The list of significantly enriched pathways from the C2, C5, and C7 of in silico analysis of GEO data comparing ccRCC to control samples, Table S3B: The list of significantly enriched pathways from the C2, C5, and C7 of A20-transfected vs. EV-transfected HEK293 cells, Table S3C: The list of significantly enriched pathways from the C2, C5, and C7 of A20-transfected vs. EV-transfected 786-O cells, Table S3D: The list of significantly enriched pathways from the C2, C5, and C7 of A20-high vs. A20-low ccRCC FFPE samples, Table S3E: The list of significantly enriched pathways in A20-high vs. A20-low ccRCC patients, Table S4A: The list of DEGs of in silico analysis of GEO data comparing ccRCC to control samples, Table S4B: The list of DEGs of A20-transfected vs. EV-transfected HEK293, Table S4C: The list of DEGs of A20-transfected vs. EV-transfected 786-O, Table S4D: The list of DEGs of A20-high vs. A20-low ccRCC FFPE samples, Table S4E: The list of DEGs A20-high vs. A20-low ccRCC patients, Table S5: Metascape pathway enrichment analysis of genes harboring variants identified by WES in A20-transfected 786-O cells.

## Abbreviations

absGSEA	absolute Gene Set Enrichment Analysis
ARHGAP6	Rho GTPase activating protein 6
BAM	Binary alignment map
ccRCC	clear cell Renal Cell Carcinoma
CARD10	Caspase recruitment domain family member 10
cDNA	Complementary DNA
CIGAR	Concise idiosyncratic gapped alignment report
CuSO_4_	Copper sulfate
CV	Coefficient of variation
DEG	Differentially expressed gene
DMEM	Dulbecco’s Modified Eagle Medium
DTT	Dithiothreitol
EdU	5-ethynyl-2′-deoxyuridine
EV	Empty Vector
FDR	False discovery rate
FFPE	Formalin-fixed paraffin-embedded
FITC	Fluorescein isothiocyanate
gcRMA	GeneChip Robust Multiarray Averaging
GEO	Gene Expression Omnibus
GPCR	G-Protein-Coupled Receptor
GSEA	Gene Set Enrichment Analysis
IFC	Integrated fluidic circuit
IGV	Integerative Genome Viewer
INDEL	Insertion/deletion
IQR	Interquartile range
IRAK1	Interleukin 1 receptor-associated kinase 1
KIRC	Kidney renal clear cell renal cell carcinoma
LUBAC	Linear ubiquitin chain assembly complex
MAS5	Microarray Suite 5
MNV	Multi-nucleotide Variant
mRNA	Messenger RNA
NEMO	NF-κB essential modulator
NGS	Next Generation Sequencing
OS	Overall Survival
OUT	Ovarian Tumor
PBS	Phosphate-buffered saline
PI	Propidium iodide
PRCC	Papillary Renal Cell Carcinoma
qPCR	Quantitative polymerase chain reaction
qRT-PCR	Quantitative Real-Time- Polymerase Chain Reaction
RCC	Renal Cell Carcinoma
RNAseq	RNA sequencing
RPMI	Roswell Park Memorial Institute
SD	Standard deviation
SDS-PAGE	Sodium dodecyl sulfate–polyacrylamide gel electrophoresis
SNV	Single-nucleotide variant
SRA	Sequence read archive
TCGA	The genome atlas
TLR	Toll-like receptor
TNF-α	Tumor Necrosis Factor-α
TNFAIP3	Tumor Necrosis Factor Alpha-induced Protein 3
UALCAN	University of Alabama at Birmingham Cancer data analysis portal
WES	Whole Exome Sequencing

## References

[1] Shembade N., Harhaj E.W. (2012). Regulation of NF-κB signaling by the A20 deubiquitinase. Cell. Mol. Immunol..

[2] Jayab N.A., Abed A., Talaat I.M., Hamoudi R. (2025). The molecular mechanism of NF-κB dysregulation across different subtypes of renal cell carcinoma. J. Adv. Res..

[3] Abbasi A., Forsberg K., Bischof F. (2015). The role of the ubiquitin-editing enzyme A20 in diseases of the central nervous system and other pathological processes. Front. Mol. Neurosci..

[4] Vereecke L., Beyaert R., van Loo G. (2009). The ubiquitin-editing enzyme A20 (TNFAIP3) is a central regulator of immunopathology. Trends Immunol..

[5] Du M.Q. (2016). MALT lymphoma: A paradigm of NF-κB dysregulation. Semin. Cancer Biol..

[6] Kato M., Sanada M., Kato I., Sato Y., Takita J., Takeuchi K., Niwa A., Chen Y., Nakazaki K., Nomoto J. (2009). Frequent inactivation of A20 in B-cell lymphomas. Nature.

[7] Honma K., Tsuzuki S., Nakagawa M., Tagawa H., Nakamura S., Morishima Y., Seto M. (2009). TNFAIP3/A20 functions as a novel tumor suppressor gene in several subtypes of non-Hodgkin lymphomas. Blood.

[8] Shi Y., Wang X., Wang J., Wang X., Zhou H., Zhang L. (2021). The dual roles of A20 in cancer. Cancer Lett..

[9] Kommer A., Meineck M., Classen P., Weinmann-Menke J. (2024). A20 in Kidney Transplantation and Autoimmunity. Int. J. Mol. Sci..

[10] Mao H., Zhao X., Sun S.C. (2025). NF-κB in inflammation and cancer. Cell. Mol. Immunol..

[11] Cooper J.T., Stroka D.M., Brostjan C., Palmetshofer A., Bach F.H., Ferran C. (1996). A20 blocks endothelial cell activation through a NF-kappaB-dependent mechanism. J. Biol. Chem..

[12] Guo Q., Jin Y., Chen X., Ye X., Shen X., Lin M., Zeng C., Zhou T., Zhang J. (2024). NF-κB in biology and targeted therapy: New insights and translational implications. Signal Transduct. Target. Ther..

[13] Jalaleddine N., Bouzid A., Hachim M., Sharif-Askari N.S., Mahboub B., Senok A., Halwani R., Hamoudi R.A., Al Heialy S. (2022). ACE2 polymorphisms impact COVID-19 severity in obese patients. Sci. Rep..

[14] Abdul Razzaq E.A., Bajbouj K., Bouzid A., Alkhayyal N., Hamoudi R., Bendardaf R. (2022). Transcriptomic Changes Associated with ERBB2 Overexpression in Colorectal Cancer Implicate a Potential Role of the Wnt Signaling Pathway in Tumorigenesis. Cancers.

[15] R Core Team (2021). R: A Language and Environment for Statistical Computing.

[16] Hamoudi R.A., Appert A., Ye H., Ruskone-Fourmestraux A., Streubel B., Chott A., Raderer M., Gong L., Wlodarska I., De Wolf-Peeters C. (2010). Differential expression of NF-kappaB target genes in MALT lymphoma with and without chromosome translocation: Insights into molecular mechanism. Leukemia.

[17] ClinVar Bethesda, MD2023. https://www.ncbi.nlm.nih.gov/clinvar/.

[18] Gouda H.R., Talaat I.M., Bouzid A., El-Assi H., Nabil A., Venkatachalam T., Bhamidimarri P.M., Wohlers I., Mahdami A., El-Gendi S. (2022). Genetic analysis of CFH and MCP in Egyptian patients with immune-complex proliferative glomerulonephritis. Front. Immunol..

[19] Robinson J.T., Thorvaldsdóttir H., Winckler W., Guttman M., Lander E.S., Getz G., Mesirov J.P. (2011). Integrative genomics viewer. Nat. Biotechnol..

[20] Presneau N., Baumhoer D., Behjati S., Pillay N., Tarpey P., Campbell P.J., Jundt G., Hamoudi R., Wedge D.C., Van Loo P. (2015). Diagnostic value of H3F3A mutations in giant cell tumour of bone compared to osteoclast-rich mimics. J. Pathol. Clin. Res..

[21] Love M.I., Huber W., Anders S. (2014). Moderated estimation of fold change and dispersion for RNA-seq data with DESeq2. Genome Biol..

[22] Penticuff J.C., Kyprianou N. (2015). Therapeutic challenges in renal cell carcinoma. Am. J. Clin. Exp. Urol..

[23] Passaro A., Al Bakir M., Hamilton E.G., Diehn M., André F., Roy-Chowdhuri S., Mountzios G., Wistuba I.I., Swanton C., Peters S. (2024). Cancer biomarkers: Emerging trends and clinical implications for personalized treatment. Cell.

[24] Ubaid S., Kushwaha R., Kashif M., Singh V. (2025). Comprehensive analysis of oncogenic determinants across tumor types via multi-omics integration. Cancer Genet..

[25] Nguyen-Tran H.H., Nguyen T.N., Chen C.Y., Hsu T. (2021). Endothelial Reprogramming Stimulated by Oncostatin M Promotes Inflammation and Tumorigenesis in VHL-Deficient Kidney Tissue. Cancer Res..

[26] Zhai H., Du Y., He H., Shen X., Hu D. (2025). WDR54 enhances NF-κB signaling to promote progression of hepatocellular carcinoma. Cancer Genet..

[27] Yu H., Lin L., Zhang Z., Zhang H., Hu H. (2020). Targeting NF-κB pathway for the therapy of diseases: Mechanism and clinical study. Signal Transduct. Target. Ther..

[28] Lee J., Cheong H. (2025). The Role of A20 in Cancer: Friend or Foe?. Cells.

[29] Mooney E.C., Sahingur S.E. (2021). The Ubiquitin System and A20: Implications in Health and Disease. J. Dent. Res..

[30] Martens A., van Loo G. (2020). A20 at the Crossroads of Cell Death, Inflammation, and Autoimmunity. Cold Spring Harb. Perspect. Biol..

[31] Kim M., Yan Y., Lee K., Sgagias M., Cowan K.H. (2004). Ectopic expression of von Hippel-Lindau tumor suppressor induces apoptosis in 786-O renal cell carcinoma cells and regresses tumor growth of 786-O cells in nude mouse. Biochem. Biophys. Res. Commun..

[32] Roberts A.M., Watson I.R., Evans A.J., Foster D.A., Irwin M.S., Ohh M. (2009). Suppression of hypoxia-inducible factor 2alpha restores p53 activity via Hdm2 and reverses chemoresistance of renal carcinoma cells. Cancer Res..

[33] Kajdaniuk D., Hudy D., Strzelczyk J.K., Młynarek K., Słomian S., Potyka A., Szymonik E., Strzelczyk J., Foltyn W., Kos-Kudła B. (2024). Transforming growth factors β and their signaling pathway in renal cell carcinoma and peritumoral space-transcriptome analysis. Clin. Transl. Oncol..

[34] Kuburich N.A., Sabapathy T., Demestichas B.R., Maddela J.J., den Hollander P., Mani S.A. (2023). Proactive and reactive roles of TGF-β in cancer. Semin. Cancer Biol..

[35] Demidova E.V., Serebriiskii I.G., Vlasenkova R., Kelow S., Andrake M.D., Hartman T.R., Kent T., Virtucio J., Rosen G.L., Pomerantz R.T. (2023). Candidate variants in DNA replication and repair genes in early-onset renal cell carcinoma patients referred for germline testing. BMC Genom..

[36] Schödel J., Grampp S., Maher E.R., Moch H., Ratcliffe P.J., Russo P., Mole D.R. (2016). Hypoxia, Hypoxia-inducible Transcription Factors, and Renal Cancer. Eur. Urol..

[37] Marquardt A., Solimando A.G., Kerscher A., Bittrich M., Kalogirou C., Kübler H., Rosenwald A., Bargou R., Kollmannsberger P., Schilling B. (2021). Subgroup-Independent Mapping of Renal Cell Carcinoma-Machine Learning Reveals Prognostic Mitochondrial Gene Signature Beyond Histopathologic Boundaries. Front. Oncol..

[38] Kastenhuber E.R., Lowe S.W. (2017). Putting p53 in Context. Cell.

[39] Gerlinger M., Rowan A.J., Horswell S., Math M., Larkin J., Endesfelder D., Gronroos E., Martinez P., Matthews N., Stewart A. (2012). Intratumor heterogeneity and branched evolution revealed by multiregion sequencing. N. Engl. J. Med..

[40] Godfrey D.I., Koay H.F., McCluskey J., Gherardin N.A. (2019). The biology and functional importance of MAIT cells. Nat. Immunol..

[41] Golonka R.M., Yeoh B.S., Saha P., Gohara A., Tummala R., Stepkowski S., Tiwari A.K., Joe B., Gonzalez F.J., Gewirtz A.T. (2023). Loss of toll-like receptor 5 potentiates spontaneous hepatocarcinogenesis in farnesoid X receptor-deficient mice. Hepatol. Commun..

[42] Peng L., He K., Cao Z., Bi L., Yu D., Wang Q., Wang J. (2020). CARD10 promotes the progression of renal cell carcinoma by regulating the NF-κB signaling pathway. Mol. Med. Rep..

[43] Turajlic S., Xu H., Litchfield K., Rowan A., Horswell S., Chambers T., O’bRien T., Lopez J.I., Watkins T.B., Nicol D. (2018). Deterministic Evolutionary Trajectories Influence Primary Tumor Growth: TRACERx Renal. Cell.

[44] Negrini S., Gorgoulis V.G., Halazonetis T.D. (2010). Genomic instability—An evolving hallmark of cancer. Nat. Rev. Mol. Cell Biol..

[45] Hanahan D., Weinberg R.A. (2011). Hallmarks of cancer: The next generation. Cell.

[46] Montúfar-Robles I., Barbosa-Cobos R.E., Romero-Díaz J., Valencia-Pacheco G., Cabello-Gutiérrez C., Ramírez-Bello J. (2024). The functional TNFAIP3 rs2230926T/G (Phe127Cys) variant confers risk to systemic lupus erythematosus in a Latin American population. Hum. Immunol..

[47] Compagno M., Lim W.K., Grunn A., Nandula S.V., Brahmachary M., Shen Q., Bertoni F., Ponzoni M., Scandurra M., Califano A. (2009). Mutations of multiple genes cause deregulation of NF-kappaB in diffuse large B-cell lymphoma. Nature.

[48] Wertz I.E., O’Rourke K.M., Zhou H., Eby M., Aravind L., Seshagiri S., Wu P., Wiesmann C., Baker R., Boone D.L. (2004). De-ubiquitination and ubiquitin ligase domains of A20 downregulate NF-kappaB signalling. Nature.

[49] Marona P., Górka J., Kwapisz O., Jura J., Rys J., Hoffman R.M., Miekus K. (2022). Resistance to tyrosine kinase inhibitors promotes renal cancer progression through MCPIP1 tumor-suppressor downregulation and c-Met activation. Cell Death Dis..

[50] Gulati S., Vogelzang N.J. (2021). Biomarkers in renal cell carcinoma: Are we there yet?. Asian J. Urol..

[51] Kruk L., Mamtimin M., Braun A., Anders H.J., Andrassy J., Gudermann T., Mammadova-Bach E. (2023). Inflammatory Networks in Renal Cell Carcinoma. Cancers.

[52] Ren N., Wang W.F., Zou L., Zhao Y.L., Miao H., Zhao Y.Y. (2023). The nuclear factor kappa B signaling pathway is a master regulator of renal fibrosis. Front. Pharmacol..

[53] Annels N.E., Denyer M., Nicol D., Hazell S., Silvanto A., Crockett M., Hussain M., Moller-Levet C., Pandha H. (2023). The dysfunctional immune response in renal cell carcinoma correlates with changes in the metabolic landscape of ccRCC during disease progression. Cancer Immunol. Immunother..

[54] Liu H., Guo Z., Wang P. (2024). Genetic expression in cancer research: Challenges and complexity. Gene Rep..

[55] Liu H., Li Y., Karsidag M., Tu T., Wang P. (2025). Technical and Biological Biases in Bulk Transcriptomic Data Mining for Cancer Research. J. Cancer.

