# Supplementary material for: Comprehensive Analysis of Genomic and Phenomic Data Reveals Context-Dependent Function of A20 (TNFAIP3) in Renal Cell Carcinoma
Source: Cancers (Basel). 2026 May 28;18(11):1775. doi: 10.3390/cancers18111775 (PMC13257325; doi:10.3390/cancers18111775)
Supplement: Supplementary file 1 [file cancers-18-01775-s001.zip › cancers-4253456 Supplementary Materials.pdf]

Supplementary Materials: Comprehensive Analysis of Genomic and Phenomic Data Reveals Context-Dependent Function of A20 (*TNFAIP3*) in Renal Cell Carcinoma

Nour Abu Jayab, Burcu Yener, Reem Sami Alhamidi, Mansi Bhavsar, Alaa Muayad Altaie, Muna Abdalla Alhammadi, Vidya Bijosh Mohan, Marwa Khamis Almazrouei, Lina Sahnoon, Rola Abujabal, Basel Al-Ramadi, Riyad Bendardaf, Iman M. Talaat and Rifat Hamoudi

A

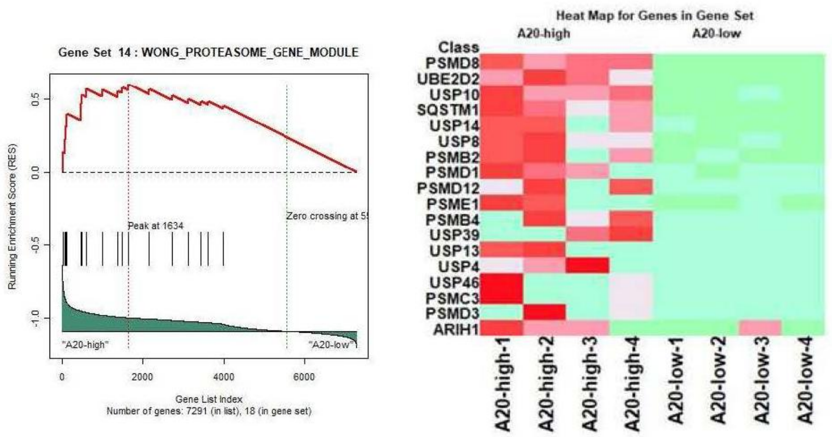

B

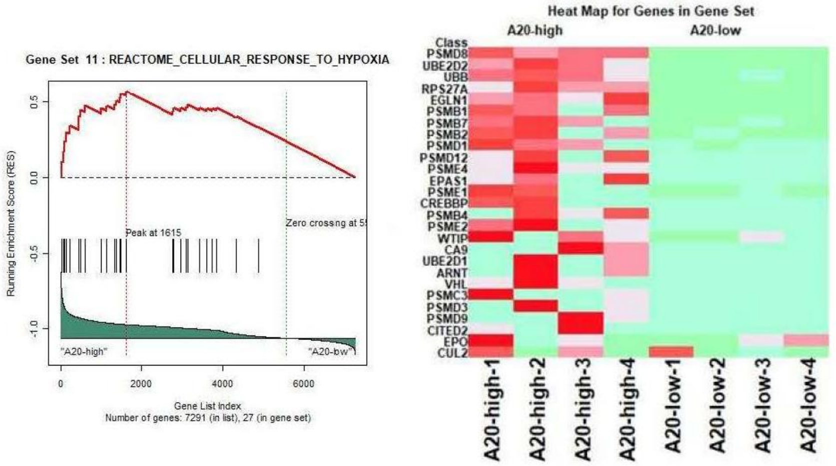

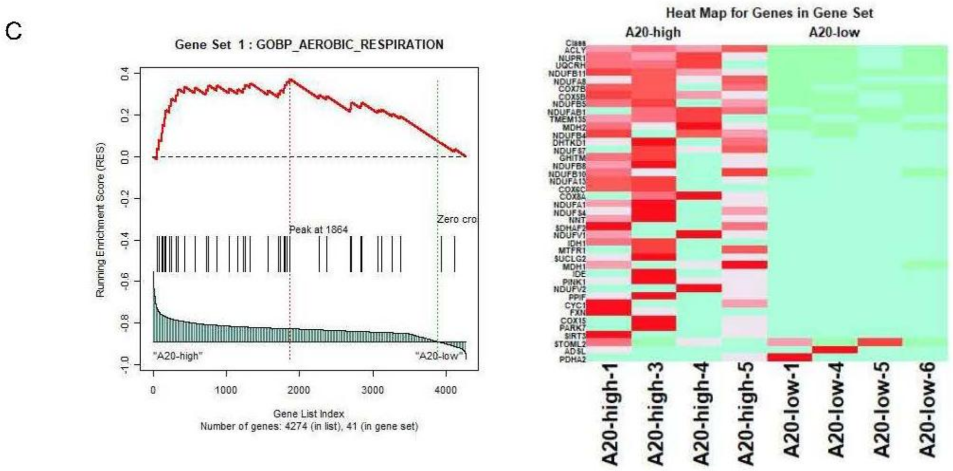

Figure S1. GSEA of enriched pathways in A20-high vs. A20-low ccRCC FFPE samples (**a**) GSEA of proteasome gene module in A20-high vs. A20-low ccRCC patients. (**b**) GSEA of cellular response to hypoxia in A20-high vs. A20-low ccRCC patients. (**c**) GSEA of aerobic respiration to hypoxia in A20-high vs. A20-low ccRCC patients.

Table S1. Demographic and clinicopathological data.

**Non-cancerous kidney samples**	
**Characteristic**	**N = 5** [1]
Age	43 (40, 46)
Sex	
Female	3 (75%)
Male	1 (25%)
Unknown	1
Diagnosis	
Hydronephrosis	1 (20%)
Pyelonephritis	4 (80%)
Nationality	
Egypt	2 (50%)
Iraq	1 (25%)
UAE	1 (25%)
Unknown	1
**PRCC**	
**Characteristic**	**N = 8**
Age	62 (58, 70)
Sex	
Female	3 (38%)
Male	5 (62%)
Unknown	0
Nuclear grade	
1	0
2	3 (38%)
3	4 (50%)
4	1 (12%)
Not applicable [1]	0
Capsular invasion	
Negative	7 (88%)

Positive	1 (12%)
Renal sinus invasion	
Negative	5 (62%)
Positive	3 (38%)
Extent	
Localized	7 (88%)
Metastatic	1 (12%)
Tumor stage	
I	3 (38%)
II	2 (25%)
III	2 (25%)
IV	1 (12%)
Systemic treatment	3 (38%)
Status	
Alive with disease	0
Died of disease	1 (12%)
Cured	7 (88%)
**ccRCC**	
**Characteristic**	**N = 8** [1]
Age	61 (51, 66)
Sex	
Female	3 (37.5%)
Male	5 (62.5%)
Diagnosis	
ccRCC	8 (100%)
Nuclear_grade	
1	1 (12.5%)
2	3 (37.5%)
3	4 (50%)
4	0 (0%)
Capsular_invasion	
Negative	5 (62.5%)
Positive	3 (37.5%)
Renal_sinus_invasion	
Negative	8 (100%)
Extent	
Localized	8 (100%)
Ki67_expression	
Negative	4 (50%)
Positive	4 (50%)
Systemic treatment	2 (25%)
Status	
Die of disease	0 (0%)
Totally cured	8 (100%)

[1] Median (IQR); n (%)

Table S2: Primer's list and sequence of DNA-targeted sequencing.

Table S3: (**A**). The list of significantly enriched pathways from the C2, C5, and C7 of in silico analysis of GEO data comparing ccRCC to control samples. (**B**) The list of significantly enriched pathways from the C2, C5, and C7 of A20-transfected vs. EV-transfected HEK293 cells. (**C**) The list of significantly enriched pathways from the C2, C5, and C7 of

A20-transfected vs. EV-transfected 786-O cells. (**D**) The list of significantly enriched pathways from the C2, C5, and C7 of A20-high vs. A20-low ccRCC FFPE samples. (**E**) The list of significantly enriched pathways in A20-high vs. A20-low ccRCC patients.

Table S4: (**A**) The list of DEGs of in silico analysis of GEO data comparing ccRCC to control samples. (**B**) The list of DEGs of A20-transfected vs. EV-transfected HEK293. (**C**) The list of DEGs of A20-transfected vs. EV-transfected 786-O. (**D**) The list of DEGs of A20-high vs. A20-low ccRCC FFPE samples. (**E**) The list of DEGs A20-high vs. A20-low ccRCC patients.

Table S5: Metascape pathway enrichment analysis of genes harboring variants identified by WES in A20-transfected 786-O cells.

Supplementary Materials Tables S2, S3, S4 and S5 can be found in separate excel files.
